# Prediction of tumor origin in cancers of unknown primary origin with cytology-based deep learning

**DOI:** 10.1038/s41591-024-02915-w

**Published:** 2024-04-16

**Authors:** Fei Tian, Dong Liu, Na Wei, Qianqian Fu, Lin Sun, Wei Liu, Xiaolong Sui, Kathryn Tian, Genevieve Nemeth, Jingyu Feng, Jingjing Xu, Lin Xiao, Junya Han, Jingjie Fu, Yinhua Shi, Yichen Yang, Jia Liu, Chunhong Hu, Bin Feng, Yan Sun, Yunjun Wang, Guohua Yu, Dalu Kong, Meiyun Wang, Wencai Li, Kexin Chen, Xiangchun Li

**Affiliations:** 1 Department of Abdominal Cancer, Tianjin’s Clinical Research Center for Cancer, Tianjin Key Laboratory of Digestive Cancer, National Clinical Research Center for Cancer, Tianjin Medical University Cancer Institute and Hospital, Tianjin Medical University, Tianjin, China; 2 Department of Radiology, The First Affiliated Hospital of Suzhou University, Suzhou, China; 3 Department of Pathology, The First Affiliated Hospital of Zhengzhou University, Zhengzhou, China; 4 Department of Pathology, Tianjin’s Clinical Research Center for Cancer, Tianjin Key Laboratory of Digestive Cancer, National Clinical Research Center for Cancer, Tianjin Medical University Cancer Institute and Hospital, Tianjin Medical University, Tianjin, China; 5 Department of Pathology, The First Affiliated Hospital of Suzhou University, Suzhou, China; 6 Department of Pathology, Yantai Yuhuangding Hospital of Qingdao University, Yantai, China; 7 Harvard Dunster House, Harvard University, Cambridge, MA USA; 8 Harvard Medical School, Boston, MA USA; 9 School of Biological Science and Medical Engineering, Beihang University, Beijing, China; 10 Tianjin Cancer Institute, Tianjin’s Clinical Research Center for Cancer, Tianjin Key Laboratory of Digestive Cancer, National Clinical Research Center for Cancer, Tianjin Medical University Cancer Institute and Hospital, Tianjin Medical University, Tianjin, China; 11 Department of Radiology, Henan Provincial People’s Hospital, The People’s Hospital of Zhengzhou University, Zhengzhou, China; 12 Department of Epidemiology and Biostatistics, Tianjin’s Clinical Research Center for Cancer, Key Laboratory of Molecular Cancer Epidemiology of Tianjin, National Clinical Research Center for Cancer, Tianjin Medical University Cancer Institute and Hospital, Tianjin Medical University, Tianjin, China

**Keywords:** Cancer screening, Metastasis, Cancer of unknown primary

## Abstract

Cancer of unknown primary (CUP) site poses diagnostic challenges due to its elusive nature. Many cases of CUP manifest as pleural and peritoneal serous effusions. Leveraging cytological images from 57,220 cases at four tertiary hospitals, we developed a deep-learning method for tumor origin differentiation using cytological histology (TORCH) that can identify malignancy and predict tumor origin in both hydrothorax and ascites. We examined its performance on three internal (*n* = 12,799) and two external (*n* = 14,538) testing sets. In both internal and external testing sets, TORCH achieved area under the receiver operating curve values ranging from 0.953 to 0.991 for cancer diagnosis and 0.953 to 0.979 for tumor origin localization. TORCH accurately predicted primary tumor origins, with a top-1 accuracy of 82.6% and top-3 accuracy of 98.9%. Compared with results derived from pathologists, TORCH showed better prediction efficacy (1.677 versus 1.265, *P* < 0.001), enhancing junior pathologists’ diagnostic scores significantly (1.326 versus 1.101, *P* < 0.001). Patients with CUP whose initial treatment protocol was concordant with TORCH-predicted origins had better overall survival than those who were administrated discordant treatment (27 versus 17 months, *P* = 0.006). Our study underscores the potential of TORCH as a valuable ancillary tool in clinical practice, although further validation in randomized trials is warranted.

## Abstract (web summary)

Developed on cytology images of hydrothorax and ascites from 57,220 cases at four hospitals, a deep-learning model shows high accuracy in tumor origin prediction and presents prognostic value when patient treatment is consistent with the cancer origin predicted by the model.

## Main

Cancers of unknown primary (CUP) site are a group of malignant diseases identified by histopathology as malignant metastases but whose origin cannot be identified by standard baseline diagnostic approaches. It is estimated that CUP accounts for 3–5% of all cancers diagnosed in humans^1–4^. Adenocarcinoma is the most common pathological type, followed by squamous and undifferentiated carcinoma^5,6^. Despite the employment of a variety of combined chemotherapies, the majority of patients have a very poor prognosis, with only 20% achieving a median survival of 10 months^7–10^. CUP are often characterized by early dissemination, aggressive clinical course and multiple organ involvement. Immunohistochemistry is usually applied as a key means of predicting its probable origin; however, less than 30% of CUP cases can be pinpointed by cocktails of approximately 20 different immunostaining subunits^7,11^ and therefore CUP remain a thorny problem for clinicians. Accurate prediction of primary sites by pathologists and oncologists is a top priority for effective and personalized treatment.

**Extended Data Fig. 1:**
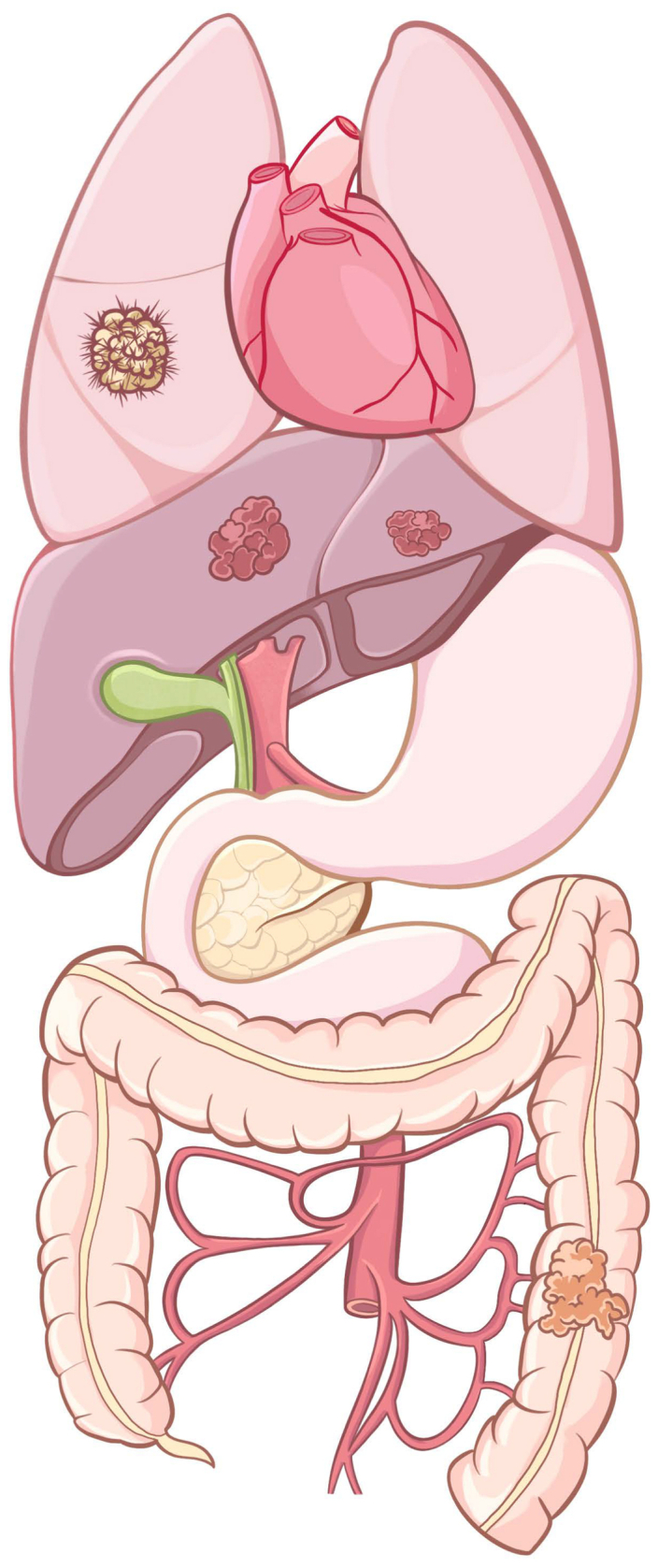
A diagram illustrating tumor metastasis. Exemplified diagram shows the tumors from chest and abdominal organs have a high possibility of malignant hydrothorax and ascites.

**Extended Data Fig. 2:**
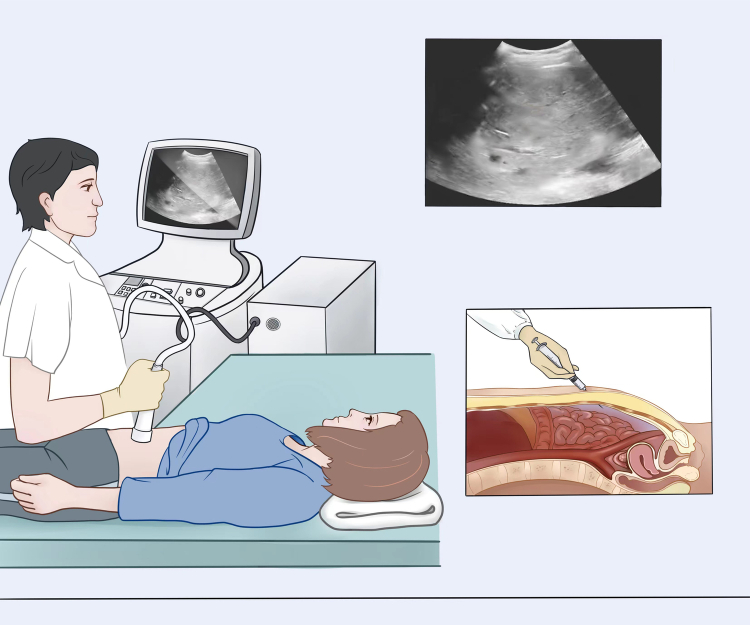
Schematic diagram of cytological examination. Hydrothorax and ascites are punctured under the guidance of color Doppler ultrasound for cytological examination.

Among patients newly diagnosed with CUP, a substantial portion present with pleural or peritoneal metastasis^7,11,12^. The thoracic and abdominal serous cavities are locations where isolated tumor cells metastasize with high proclivity (Extended Data Fig. 1). Free tumor cells or implanted clusters found in pleural effusion or ascites are strong evidence of stage IV for some solid tumors^13–16^. It has been reported that 7–20% of patients with respiratory or gastrointestinal tumors are diagnosed with pleural and peritoneal effusions, many of whom have synchronous peritoneal or pleural carcinomatosis^13–19^. Previous studies revealed that serous effusions may develop without any history of cancer and present as the initial manifestation of cancer in 10% of patients with malignant effusions^20–23^. Cytological examination by peritoneal or pleural fine-needle aspiration is usually used as a key method in the diagnosis of thoracoabdominal metastasis (Extended Data Fig. 2)^24–26^. Most often, however, pathologists can visually distinguish adenocarcinoma from squamous carcinoma on cytology smears, but not the origin of the tumor cells^13,23,25^. Therefore, precise cytological assessment may help in the appropriate management of patients with CUP and pleural or peritoneal metastasis, guide optimal therapeutic strategies, avoid unnecessary surgeries and further prolong overall survival^27–29^.

Computerized analysis based on deep convolutional neural networks has recently been increasingly applied as an auxiliary technique in the field of pathological diagnosis^30–32^. Digital pathology has been applied to a variety of image-processing and image-classification tasks, including low-level object recognition and high-level disease prognosis or treatment-response prediction. Previous studies have reported the on-par performance of artificial intelligence (AI) models as compared with pathologists in the detection of breast cancer lymph node metastases, prediction of prostate cancer Gleason grading and interpretion of the likelihood of gastric cancer^33–35^. Lu et al. also reported an AI model that showed potential benefits as a diagnostic assistive tool for CUP origin prediction using whole-slide images^36^. However, these algorithms focused mainly on histological or whole-slide images; a deep-learning model that can interpret cytological imaging data to predict tumor origin is rarely reported^37^. In routine clinical practice, histological and cytological pathologies have different application scenarios in terms of the tracking of tumor origin. Histological examination is used when specimens can be obtained via surgery or needle biopsy, these types of specimen providing richer diagnostic information. Cytology is mainly applicable for patients with late-stage cancer who cannot undergo surgery or tolerate needle biopsy^25,38^. In this scenario, specimens from pleural and peritoneal serous effusion are helpful in regard to localization of cancer origins due to their excellent accessibility^26,39^. However, sampling inadequacy (low cellular harvest), cellular degeneration or atypia and interexaminer variation in interpretation are major reasons for suboptimal diagnostic accuracy^25,39,40^. Application of new techniques is required, such as AI auxiliary image analysis, to improve tumor detection capability. To the best of our knowledge, employment of AI in the prediction of cancer origin using cytological images from hydrothorax and ascites has not been investigated.

In this study we aimed to establish a diagnostic model to predict the broad cancer origins in patients with cancer and hydrothorax or ascites metastasis using cytological images. The performance of our AI system is examined and validated by large-scale cytological smear cases from several independent testing sets.

## Results

### Baseline characteristics of patients and image datasets

**Fig. 1:**
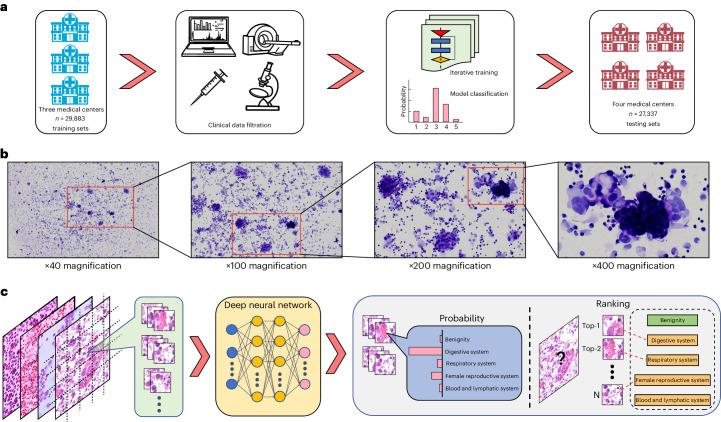
Our proposed TORCH model framework. **a**, A total of 42,682 cases were sourced from three large tertiary referral institutions, 70% of which (*n* = 29,883) were used as training sets. Clinicopathological data were acquired from radiological imaging departments, medical records systems and pathological digital databases. **b**, During the diagnostic process, most images were magnified either ×200 or ×400. **c**, The deep-learning network, trained with cytological images, was aimed at dividing target images into five categories according to the highest predicted probability score. Classification results were further validated at four institutions, including three internal testing sets (*n* = 12,799) and two external testing sets (*n* = 14,538). N represents the N-th image tile.

**Extended Data Fig. 3:**
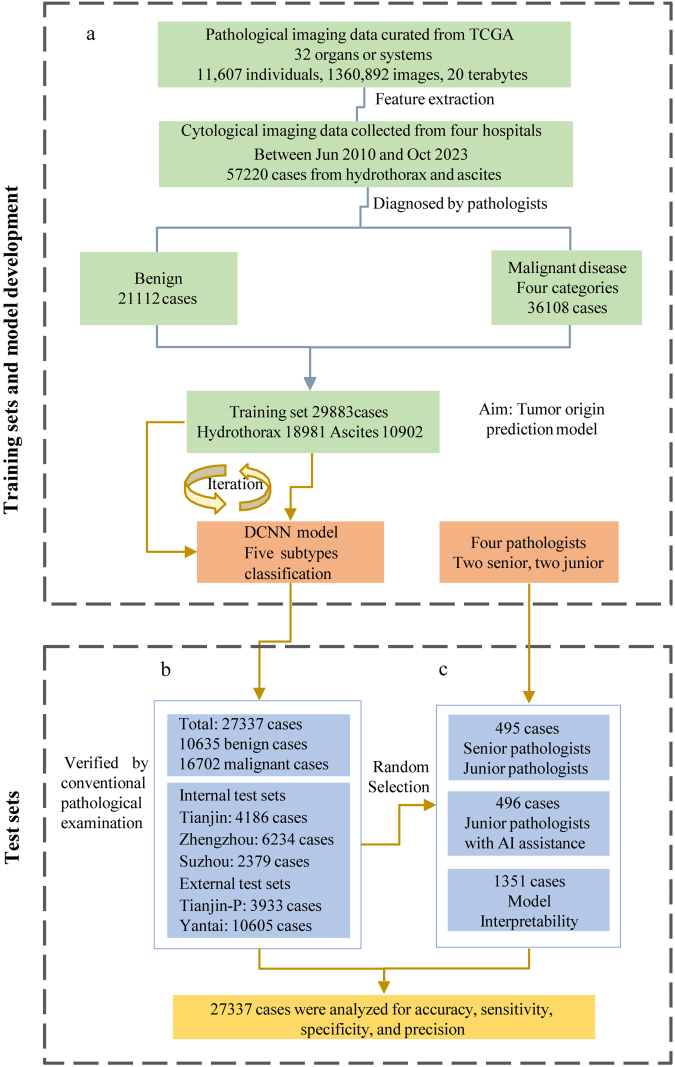
The flowchart exhibiting the procedures to develop and evaluate TORCH model. **a**, Model development procedure consisted of feature extraction, real clinical data taxonomy and model iteration. **b**, Evaluation of TORCH on three internal and two external testing sets. **c**, Performance comparison between TORCH and four pathologists on randomly selected cases.

Between June 2010 and October 2023 we obtained a large dataset of 90,572 cytological smear images from 76,183 patients at four large institutions (Tianjin Medical University Cancer Institute and Hospital, Zhengzhou University First Hospital, Suzhou University First Hospital and Yantai Yuhuangding Hospital) as the training and testing sets (Table 1). We excluded 24,808 malignancy images lacking any clinical or pathological supporting evidence for the primary origins. A further 8,544 blank or poorly focused images were also excluded. The ultimate dataset consisted of 57,220 images from 43,688 patients (Extended Data Fig. 3). The training set consisted of 29,883 images from 20,638 individuals covering 12 tumor subtypes or origins: 138, esophagus; 1,773, stomach; 20, intestine; 720, colon and rectum; 151, liver; 144, gallbladder; 357, pancreas; 321, uterus and vagina; 4,217, ovary and fallopian tube; 1,874, breast; 9,121, lung and upper respiratory tract; and 570, blood and lymphatic system. In addition to the 19,406 tumor images described above, 10,477 images of benign diseases were also included in the final training set. Similarly, three internal testing sets comprising 10,974 individuals (12,799 images) were obtained from the same four hospitals. Two additional external testing sets comprised 12,076 individuals (14,538 images) from Tianjin and Yantai hospitals (Fig. 1). The tumor category of testing sets was broadly in line with that of the training set. Because one patient might have undergone more than one hydrothorax or ascites core needle biopsy for cytological analysis at various stages of disease development, more than one image may have been recorded. In this study, each image combined with its clinicopathological data was compiled as one case. Respiratory diseases accounted for the largest proportion (29.8%, *n* = 17,058) among malignant groups. Carcinoma amounted to 56.7% (*n* = 32,424) of overall hydrothorax and ascites cytological cases, among which adenocarcinoma comprised the largest category (47.2%, *n* = 27,006). The proportion of squamous cell carcinoma metastasizing to pleural effusion or ascites was only 0.6% (*n* = 346). In addition, there were 24,658 (82.5%) cases in the training set stratified as high certainty and 5,225 (17.5%) as low certainty. For the testing sets, 18,184 (66.5%) cases were stratified as high certainty and 9,153 (33.5%) as low certainty. With respect to images of malignancy, 6,066 of 19,406 (31.2%) cases in the training set and 4,256 of 16,702 (25.5%) cases in the testing sets also underwent examination by sediment paraffin immunohistochemical staining.

**Table 1:** Baseline characteristics of training and testing sets

Parameter	Overall, *n* = 57,220 (%)	Training sets (*n* = 29,883)			Internal testing sets (*n* = 12,799)			External testing sets (*n* = 14,538)	
		Tianjin, *n* = 9,822 (%)	Zhengzhou, *n* = 14,586 (%)	Suzhou, *n* = 5,475 (%)	Tianjin, *n* = 4,186 (%)	Zhengzhou, *n* = 6,234 (%)	Suzhou, *n* = 2,379 (%)	Tianjin-P^c^, *n* = 3 933 (%)	Yantai, *n* = 10,605 (%)
Male sex	25,822 (45.1)	3,223 (32.8)	7,353 (50.4)	2,862 (52.3)	1,369 (32.7)	3,111 (49.9)	1,235 (51.9)	1,792 (45.6)	4,877 (46.0)
Female sex	31,398 (54.9)	6,599 (67.2)	7,233 (49.6)	2,613 (47.7)	2,817 (67.3)	3,123 (50.1)	1,144 (48.1)	2,141 (54.4)	5,728 (54.0)
Age, years (mean ± SD)	59.13 ± 14.21	58.23 ± 11.47	57.17 ± 16.03	63.02 ± 14.40	58.25 ± 11.54	57.15 ± 15.94	63.57 ± 14.09	60.27 ± 12.58	60.73 ± 13.32
Age ≤60 years	28,079 (49.1)	5,386 (54.8)	7,702 (52.8)	2,019 (36.9)	2,284 (54.6)	3,287 (52.7)	854 (35.9)	1,797 (45.7)	4,750 (44.8)
Age >60 years	29.141 (50.9)	4,436 (45.2)	6,884 (47.2)	3,456 (63.1)	1,902 (45.4)	2,947 (47.3)	1,525 (64.1)	2,136 (54.3)	5,855 (55.2)
Primary tumor site									
Digestive	5,682 (9.9)	1,504 (15.3)	1,135 (7.8)	664 (12.1)	591 (14.1)	544 (8.7)	270 (11.3)	315 (8.0)	659 (6.2)
Female reproductive	12,350 (21.6)	3,901 (39.7)	1,772 (12.1)	739 (13.5)	1,662 (39.7)	774 (12.4)	331 (13.9)	888 (22.6)	2,283 (21.5)
Respiratory	17,058 (29.8)	3,239 (33.0)	3,742 (25.7)	2,140 (39.1)	1,433 (34.2)	1,589 (25.5)	958 (40.3)	1,135 (28.9)	2,822 (26.6)
Blood and lymphatic	1,018 (1.8)	73 (0.7)	417 (2.9)	80 (1.5)	30 (0.7)	214 (3.4)	33 (1.4)	63 (1.6)	108 (1.0)
Benign	21,112 (36.9)	1,105 (11.3)	7,520 (51.6)	1,852 (33.8)	470 (11.2)	3,113 (49.9)	787 (33.1)	1,532 (39.0)	4,733 (44.6)
Hydrothorax	35,873 (62.7)	5,751 (58.6)	9,427 (64.6)	3,803 (69.5)	2,491 (59.5)	4,031 (64.7)	1,637 (68.8)	2,364 (60.1)	6,369 (60.1)
Ascites	21,347 (37.3)	4,071 (41.4)	5,159 (35.4)	1,672 (30.5)	1,695 (40.5)	2,203 (35.3)	742 (31.2)	1,569 (39.9)	4,236 (39.9)
Carcinoma	32,424 (56.7)	7,944 (80.9)	5,250 (36.0)	3,203 (58.5)	3,670 (87.7)	2,900 (46.5)	1,547 (65.0)	2,335 (59.4)	5,575 (52.6)
Adenocarcinoma	27,006 (47.2)	7,218 (73.5)	4,279 (29.3)	2,622 (47.9)	3,022 (72.2)	1,851 (29.7)	1,129 (47.5)	2,056 (52.3)	4,829 (45.5)
Squamous carcinoma	346 (0.6)	50 (0.5)	130 (0.9)	30 (0.5)	24 (0.6)	60 (1.0)	23 (1.0)	7 (0.2)	22 (0.2)
Other carcinoma^a^	1,518 (2.7)	166 (1.7)	294 (2.0)	207 (3.8)	53 (1.3)	111 (1.8)	88 (3.7)	185 (4.7)	414 (3.9)
Unclassified^b^	3,554 (6.2)	510 (5.2)	547 (3.8)	344 (6.3)	571 (13.6)	878 (14.1)	307 (12.9)	87 (2.2)	310 (2.9)
High-certainty cases	42,912 (75.0)	9,355 (95.2)	10,708 (73.4)	4,595 (83.9)	3,992 (95.4)	4,270 (68.5)	1,981 (83.3)	2,052 (52.2)	5,959 (56.2)
Low-certainty cases	14,308 (25.0)	467 (4.8)	3,878 (26.6)	880 (16.1)	194 (4.6)	1,964 (31.5)	398 (16.7)	1,881 (47.8)	4,646 (43.8)

^a^Other types consist mainly of sarcomatoid carcinoma, adenosquamous carcinoma, papillary carcinoma, large cell carcinoma, small cell carcinoma, transitional epithelial carcinoma, basal cell carcinoma and undifferentiated carcinoma.

^b^Unclassified carcinoma means that the specific type of cancer is unknown, the main reason being that the specimen was too small for staining by immunohistochemistry.

^c^Tianjin-P, Tianjin external testing set enrolled prospectively.

### Performance of TORCH on prediction of tumor origin

**Fig. 2:**
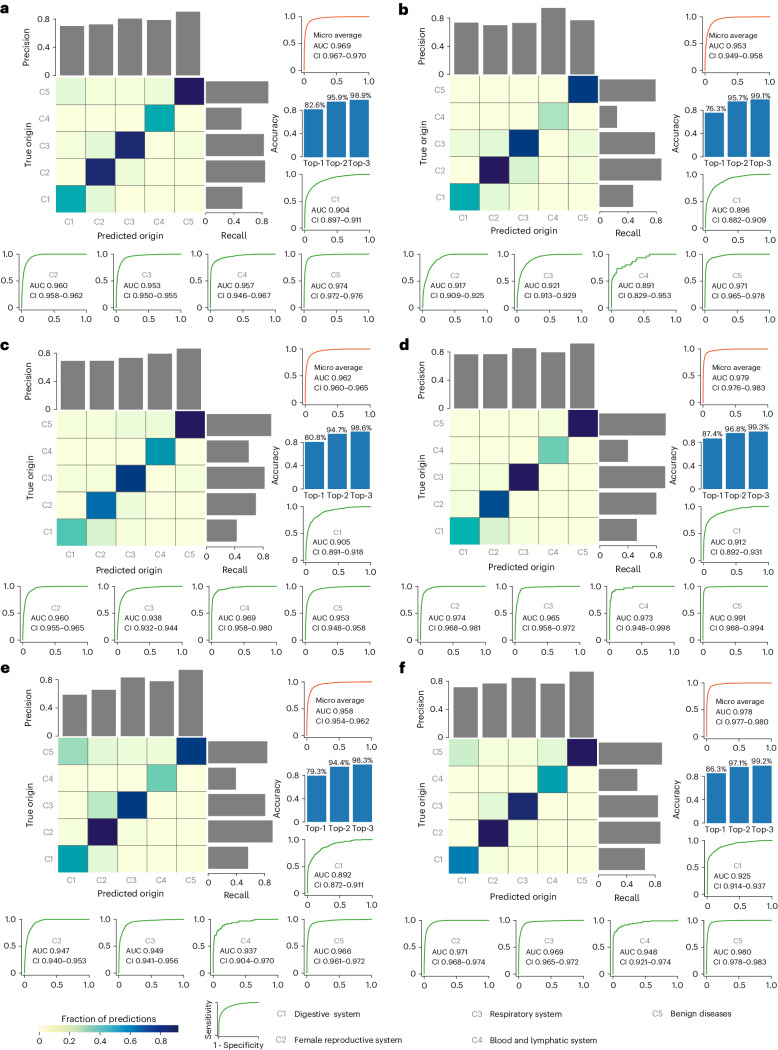
Classification performance of the TORCH model. **a**, The confusion matrix, including precision and recall, is plotted for prediction of isolated tumor cell origin on the overall five testing sets (*n* = 27,337). Microaveraged one-versus-rest ROC curves for the five categories (red curves). Top-*n* model (*n* = 1, 2, 3) accuracy for tumor origin classification. **b**–**f**, Five ROC curves for the auxiliary binary task of prediction of malignancy or benignity and prediction of four tumor categories (green curves). **b**, Tianjin testing set. **c**, Zhengzhou testing set. **d**, Suzhou testing set. **e**, Tianjin-P testing set. **f**, Yantai testing set. AUC, area under the curve.

We developed TORCH by training four different deep neural networks on three different types of input, giving rise to 12 different models (Methods). The classification results of each individual model are shown in Supplementary Figs. 1–4 and Supplementary Tables 1–4. We subsequently performed model ensembling to integrate these models (Methods). The results showed that TORCH provides relatively reliable generalization and interoperability. On the five testing sets (*n* = 27,337), TORCH achieved an overall microaveraged one-versus-rest area under the receiver operating characteristic (AUROC) value of 0.969 (95% confidence interval (CI) 0.967–0.970). On the three internal testing sets, microaveraged one-versus-rest AUROC values were 0.953 (CI 0.949–0.958) for the Tianjin dataset, 0.962 (CI 0.960–0.965) for the Zhengzhou dataset and 0.979 (CI 0.976–0.983) for the Suzhou dataset (Fig. 2). On the two external testing sets, microaveraged one-versus-rest AUROC values were 0.958 (CI 0.954–0.962) and 0.978 (CI 0.977–0.980) for the Tianjin-P and Yantai datasets, respectively. In terms of identification of cancer-positive cases, TORCH achieved an AUROC value of 0.974 (CI 0.972–0.976), accuracy of 92.6% (CI 92.2–92.9%), sensitivity of 92.8% (CI 92.3–93.2%) and specificity of 92.4% (CI 92.0–92.8%) (Extended Data Table 1). In terms of tumor origin localization in the female reproductive system group, TORCH achieved an AUROC value of 0.960 (CI 0.958–0.962), accuracy of 88.1% (CI 87.7–88.5%), sensitivity of 92.5% (CI 91.8–93.2%) and specificity of 86.9% (CI 86.4–87.3%), an enhanced performance compared with that for the other systems. In addition, the effectiveness of this model was stable in that it achieved similar results among the five testing sets. Detailed classification metrics of the five categories are provided in Extended Data Table 2 and Supplementary Table 5. The model prediction results of 27,337 cases are shown in Supplementary Table 6.

TORCH achieved a top-1 accuracy of 82.6%, top-2 accuracy of 95.9% and top-3 accuracy of 98.9% when combining these five testing sets. These top-*n* accuracies fluctuated within a narrow range among the five testing sets (Fig. 2). On the Tianjin internal testing set (*n* = 4,186), the top-*n* accuracies achieved by TORCH were 76.3, 95.7 and 99.1%, respectively; on the Zhengzhou testing set (*n* = 6,234), these were 80.8, 94.7 and 98.6%, respectively; and on the Suzhou testing set (*n* = 2,379), these were 87.4, 96.8 and 99.3%, respectively. With respect to stratification by specimen sampling site, TORCH achieved higher microaveraged one-versus-rest AUROC (0.970 (CI 0.969–0.972)) in the hydrothorax group than in the ascites group (0.966 (CI 0.964–0.969; *P* < 0.001); Supplementary Fig. 5 and Supplementary Table 7). Among the five categories, TORCH achieved higher AUROC values in ascites than in hydrothorax for the digestive (0.892 versus 0.775, *P* < 0.001) and female reproductive systems (0.951 versus 0.945, *P* = 0.012) and lower AUROC values for the respiratory system (0.808 versus 0.929, *P* < 0.001). No significant differences were observed for benign diseases (0.972 versus 0.975, *P* = 0.068) or the blood and lymphatic system (0.967 versus 0.951, *P* = 0.122) in ascites versus hydrothorax. In addition, when solid tumors were divided into carcinoma and noncarcinoma, we observed that TORCH achieved comparable AUROC values in both the carcinoma group (0.938 (CI 0.936–0.940)) and the noncarcinoma group (0.939 (CI 0.921–0.958); *P* = 0.244). Within the carcinoma group, TORCH exhibited slightly better performance for the adenocarcinoma group versus the nonadenocarcinoma group (AUROC, 0.942 (CI 0.939–0.944) versus 0.925 (CI 0.919–0.931) (*P* = 0.002)).

**Extended Data Fig. 4:**
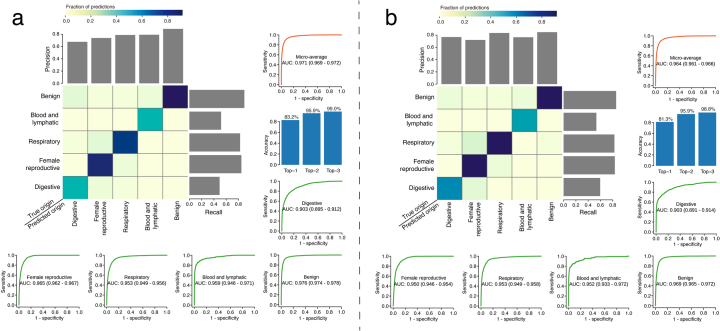
Classification performance of TORCH model on high-certainty cases and low-certainty cases respectively. Overall micro-averaged one-versus-rest auroc is similar for cases in the low-certainty group (**b**) compared with high-certainty group (**a**) [0.964 (0.961–0.966) versus 0.971 (0.969–0.972) (P = 0.106)].

To explore TORCH further we examined its prediction efficiency on both high- and low-certainty cases. TORCH achieved comparable microaveraged one-versus-rest AUROC values in the low-certainty group compared with the high-certainty group (0.964 (CI 0.961–0.966) versus 0.971 (CI 0.969–0.972), *P* = 0.106; Extended Data Fig. 4). Meanwhile, no significant difference in terms of classification metrics was observed between the two subgroups. Classification metrics including accuracy, sensitivity, specificity, precision and negative predictive value are shown in Supplementary Table 8.

To further verify the generalization and reliability of TORCH, we enrolled 4,520 consecutive cases from Tianjin Cancer Hospital (the Tianjin-P dataset) and 12,467 from Yantai Hospital (the Yantai dataset) as fully unseen external testing sets. These images were collected from pathological databases without exclusion of any cases. The Tianjin-P and Yantai datasets included 587 and 1,862 uncertainty cases, respectively. We observed that TORCH achieved top-1/2/3 accuracy of 79.3, 94.4 and 98.3%, respectively, on the Tianjin-P dataset without uncertainty cases (*n* = 3,933) and 86.3, 97.1 and 99.2%, respectively, on the Yantai dataset without uncertainty cases (*n* = 10,605). The lower-bound top-1 accuracy of TORCH was estimated to be 70.2% on the Tianjin-P dataset and 75.1% on the Yantai dataset by assuming that all predictions made by TORCH for these uncertainty cases were erroneous. The upper-bound top-1 accuracy of TORCH was estimated to be 81.7% on the Tianjin-P dataset and 88.1% on the Yantai dataset by assuming that all predictions made by TORCH for these uncertainty cases were correct.

### Performance of TORCH versus pathologists

**Fig. 3:**
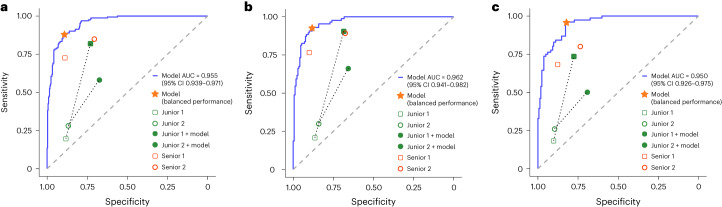
Comparison of diagnostic performance of pathologists and TORCH in differentiating benign from malignant samples. **a**–**c**, Both the TORCH model and senior pathologists demonstrated higher sensitivity than junior pathologists in differentiating benign from malignant samples in the entire test subset (**a**), hydrothorax subset (**b**) and ascites subset (**c**). The four pathologists’ original performances are denoted by unfilled dots, and those of the junior pathologists with TORCH assistance by filled dots. Dashed lines connect paired performance points of the two junior radiologists. The star denotes the performance of TORCH in the ‘balanced performance’ setting.

We asked two junior and two senior practicing pathologists to manually interpret 495 cytological images that comprised 333 malignant cases and 162 benign cases, with subsequent comparison with predictions made by TORCH. We observed that top-1 accuracies were 42.6% (95% CI 38.2–46.9%) and 44.0% (95% CI 39.4–47.9%) for the two junior pathologists and 69.7% (95% CI 66.3–73.5%) and 57.0% (95% CI 52.9–61.2%) for the two senior pathologists. Notably, TORCH achieved a top-1 accuracy of 78.8% (95% CI 75.4–82.0%), which was significantly higher than that for the four pathologists (permutation test, all *P* < 0.001). When stratified by the five categories, TORCH outperformed pathologists with respect to accuracy (mean 0.896 versus 0.813; *P* = 0.038), sensitivity (mean 0.880 versus 0.485; *P* < 0.001) and precision (mean 0.634 versus 0.486; *P* < 0.001; Extended Data Table 3). TORCH also achieved marginally higher specificity compared with this group of pathologists, although the difference did not reach statistical significance (mean 89.4% versus 87.8%; *P* = 0.333). Receiver operating characteristic (ROC) curves of TORCH for the five categories of these 495 cases are provided in Supplementary Fig. 6. TORCH achieved significantly higher diagnostic scores compared with the pathologists (1.677 (95% CI 1.647–1.706) versus 1.265 (95% CI 1.227–1.302), *P* < 0.001). The senior pathologists also achieved higher diagnostic scores compared with the junior pathologists (1.428 (95% CI 1.378–1.479) versus 1.101 (95% CI 1.047–1.155), *P* < 0.001; Supplementary Table 9). Inter-rater agreement rate for the four pathologists was 24.6% (122 of 495, Fleiss’ kappa 0.365, two-sided *z*-test, *P* < 0.001). Although inter-rater agreement rate was statistically significant, it was still relatively low among the pathologists and could be considered to be in fair agreement according to Landis and Koch^41^. This suggested that interpretation of cytological images for assessment of tumor origin is subject to substantial variability. In addition, the senior pathologists achieved significantly higher performance compared with their junior counterparts in terms of both accuracy (0.853 versus 0.773, *P* = 0.014) and precision (0.594 versus 0.381, *P* = 0.001; Supplementary Table 9). In addition, both TORCH and the senior pathologists recorded higher sensitivity than the junior pathologists in differentiation of benign diseases from malignant tumors (Fig. 3). The performances of both senior and junior pathologists are shown in Supplementary Tables 9–12 and Supplementary Fig. 6.

### Performance of pathologists with TORCH assistance

To determine whether the performance of the junior pathologists could be improved with the assistance of TORCH, an additional 496 cytology smear images (not overlapping with the 495 cytological images) were randomly selected from three internal testing sets. TORCH-predicted tumor origins were presented to these two junior pathologists for reference and they were asked to perform differential diagnosis independently. Subsequently the performance of these junior pathologists, with the assistance of TORCH, was compared with previous manual interpretation results for both junior and senior pathologists. We observed that the junior pathologists with the assistance of TORCH achieved significantly higher overall top-1 accuracy than without TORCH (62.3% (95% CI 59.3–64.9%) versus 43.3% (40.0–46.0%); permutation test, *P* < 0.001), and achieved top-1 accuracy comparable to that of senior pathologists (63.3% (95% CI 60.3–66.1%); permutation test, *P* = 0.777). Top-2/3 accuracies were not available for pathologists. Among these five categories, when assisted by TORCH, the accuracy of these two junior pathologists in regard to the digestive system was improved the most (*P* = 0.032), increasing from 78.8% (74.9–82.3%) to 89.3% (86.3–91.9%) and from 79.0% (75.1–82.5%) to 88.5% (85.4–91.2%), respectively. In terms of sensitivity, the classification efficacy of the two junior pathologists in regard to the female reproductive system was markedly improved (from 63.4% (CI 53.8–72.3) to 80.0% (CI 66.3–90.0) and from 65.2% (CI 55.6–73.9) to 84.0% (CI 70.9–92.8), *P* = 0.039). In regard to differentiation of benign diseases and malignant tumors, the performance of the junior pathologists was improved substantially, with a marked increase in sensitivity (Fig. 3 and Extended Data Table 4). Meanwhile, the mean diagnostic score for the junior pathologists with the assistance of TORCH was significantly higher than without TORCH (1.326 (95% CI 1.269–1.382) versus 1.101 (95% CI 1.047–1.155); *P* < 0.001). The classification performance of junior pathologists with AI assistance is shown in Extended Data Table 4, Supplementary Table 13 and Supplementary Fig. 7. Although the diagnostic efficacy of junior pathologists was improved with the assistance of TORCH, their diagnostic score was still lower than that of TORCH itself (1.326 (95% CI 1.269–1.382) versus 1.829 (CI 1.785–1.872); *P* < 0.001). Meanwhile, TORCH-assisted junior pathologists did not reach the same level as the senior pathologists (1.326 (95% CI 1.269–1.382) versus 1.428 (CI 1.378–1.479); *P* = 0.008). Detailed diagnostic scores are provided in Supplementary Table 14.

### Ablation results

The inputs to TORCH include both imaging and clinical data modalities. Because clinical parameters such as age, sex and specimen sampling site are often considered auxiliary in the assessment of tumor origin, we therefore removed these in our ablation study. Results showed that ablation of sex, age and specimen sampling site led to a substantial decrease in both AUROC and accuracy. We observed that there were significant decreases in microaveraged one-versus-rest AUROC values (0.969 versus 0.925, *P* < 0.001), top-1 accuracy (82.6% versus 68.9%, *P* < 0.001) and top-2 accuracy (95.9% versus 88.7%, *P* < 0.001). Among these five categories on the combined dataset, AUROC values were also significantly decreased for the digestive system (0.904 versus 0.803, *P* < 0.001), female reproductive system (0.960 versus 0.841, *P* < 0.001), respiratory system (0.953 versus 0.838, *P* < 0.001), blood and lymphatic system (0.957 versus 0.946, *P* < 0.001) and benign diseases (0.974 versus 0.972, *P* = 0.020). This suggests that the ability of the TORCH model in regard to origin prediction actually acquired benefits from merging of these three basic parameters. Confusion matrices, precision, recall rate and other classification metrics of TORCH with ablation are presented in Supplementary Fig. 8 and Supplementary Tables 15 and 16. To assess the impact of relationships between clinical variables and cytological imaging on model performance, we randomly perturbed clinical variables and subsequently compared differences in performance with and without perturbation of clinical variables (Methods). On the combined overall dataset we observed that *∆*^age^ = 6.70%, *∆*^sex^ = 26.5% and *∆*^site^ = 37.5%. This suggested that specimen sampling site has the highest impact, followed by sex and age.

### TORCH prediction and therapy response

**Fig. 4:**
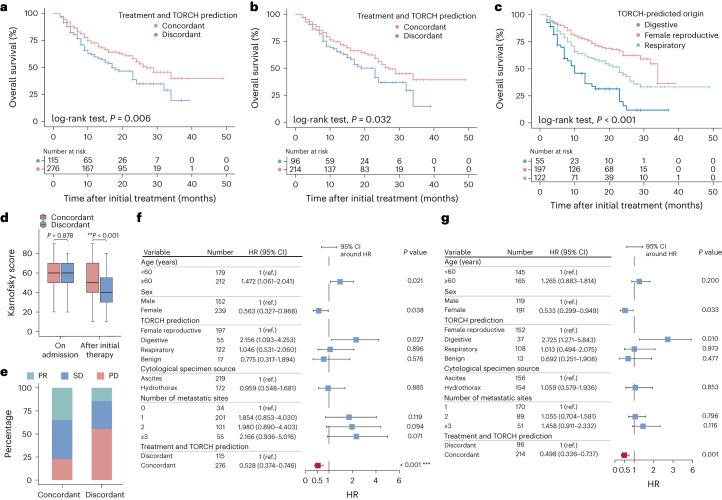
Correlation between TORCH prediction and long-term outcome of patients with CUP. **a**–**g**, A cohort of 391 patients with CUP, defined as uncertainty cases, was retrospectively collected: 276 were categorized as the concordant group and 115 as the discordant group; 310 patients (214 in the concordant group, 96 in the discordant group) received palliative chemotherapy and targeted drugs combined with or without radiotherapy. **a**,**b**, Kaplan–Meier survival curves of overall survival for 391 (**a**) and 310 patients (**b**) with CUP. Red line, concordant group; blue line, discordant group. **c**, TORCH-predicted tumor origin as digestive system for 55 patients with CUP, female reproductive system origin for 197 and respiratory system for 122. Patients with a tumor of the female reproductive system origin showed significantly better overall survival than the other two groups (*P* = 2.2 × 10^−16^). **d**, Between 3 and 6 months after initial treatment, Karnofsky score for patients in the concordant group (*n* = 276) was significantly higher than that for the discordant group (*n* = 115; 52.1 ± 18.8 versus 41.8 ± 19.5, two-sided Student’s *t*-test, **P = 2.818 × 10^−6^). Adjustment for multiple comparisons was conducted for the tests at the timepoints of admission and after initial therapy using Bonferroni correction. The upper bar represents maxima, the lower bar minima; the upper bound of the box represents 75% site value, the lower bound 25%; the upper whisker contains 25% high-value data, the lower whisker 25% low-value data; the horizontal line in the middle of the box represents the median. **e**, Of the 310 patients, the percentages of clinical PR, SD and PD in the concordant group were 35.0 (75 of 214), 42.5 (91 of 214) and 22.4 (48 of 214), respectively; correspondingly, the percentages of clinical PR, SD and PD in the discordant group were 14.6 (14 of 96), 30.2 (29 of 96) and 55.2 (53 of 96), respectively. **f**,**g**, Multivariate Cox regression analysis indicated that concordance (red box) is an independent favorable factor for better prognosis. **f**, The cohort of 391 CUP patients defined as uncertainty cases that were treated by palliative chemotherapy, targeted drugs, surgery and supportive regimens. Two-sided Cox proportional-hazards test, *n* = 391, HR 0.528, 95% CI 0.374–0.746, ***P = 2.91 × 10^−4^. **g**, 310 CUP patients out of the above 391 CUP patients who received palliative chemotherapy and targeted drugs. Two-sided Cox proportional-hazards test, *n* = 310, HR 0.498, 95% CI 0.336–0.737, *P* = 0.001. Bars represent 95% CI of HR; blue and red boxes represent the value of HR.

To determine whether clinical benefits were achieved for patients with CUP who received treatment in concordance with TORCH-predicted cancer origin, we performed survival analysis for 391 of these patients. Certified oncologists reviewed their hospitalization records to determine whether their treatments were concordant with TORCH-predicted cancer origins (Methods). Of these 391 patients, 276 and 115 were categorized into the concordant and discordant groups, respectively. At the end of follow-up 163 (41.7%) patients had died: 102 (36.9%) in the concordant group and 61 (53.0%) in the discordant group. Kaplan–Meier survival analysis showed that the concordant group had significantly better overall survival compared with the discordant group (median overall survival 27 months (95% CI 25–34) versus 17 months (95% CI 15–23); log-rank test, *P* = 0.006; Fig. 4). Specifically, patients whose tumor was predicted to be of digestive system origin had a worse prognosis compared with those whose cancer origin was predicted to be the respiratory or female reproductive system (*P* < 0.001; Fig. 4). At 3–6 months after initial treatment, Karnofsky score was significantly lower in the discordant group than in the concordant group (41.8 ± 19.5 versus 52.1 ± 18.8, *P* < 0.001). In addition, clinical benefits were further evaluated according to Response Evaluation Criteria In Solid Tumors criteria. For those 310 patients who underwent palliative chemotherapy or targeted drugs, in the concordant group 75 achieved clinical partial response (PR) by imaging evaluation, 91 achieved stable disease (SD) and 48 demonstrated progressive disease (PD). In the discordant group, 14 patients achieved PR, 29 achieved SD and 53 demonstrated PD. No patient achieved complete response in our study. With regard to these 310 patients, those in the concordant group (*n* = 214) also exhibited significantly better overall survival compared with the discordant group (*n* = 96) (*P* = 0.032). Covariates including age, sex, AI prediction type, cytological specimen source, metastatic site number and concordance were analyzed by stepwise Cox proportional-hazards model. Multivariate Cox regression analysis indicated that concordance was an independent favorable factor for better prognosis (group with 391 patients: hazard ratio (HR) 0.528, 95% CI 0.374–0.746, *P* < 0.001; group with 310 patients: HR 0.498, 95% CI 0.336–0.737, *P* = 0.001; Fig. 4). Detailed clinical characteristics, treatment plan and survival data on these 391 patients are provided in Supplementary Table 17.

### Analysis of false results

**Fig. 5:**
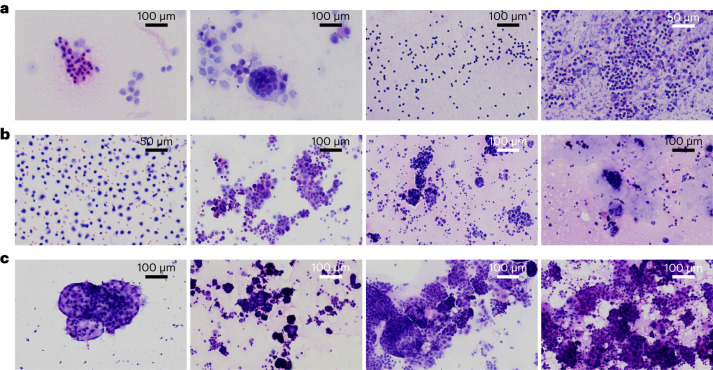
Exemplified cytological images of several characteristic cancer and benign specimens. **a**, Falsely classified benign cases, from left to right: beaded degenerated histocytes misidentified as female reproductive system (×200); reactive hyperplasia-aggregated mesothelial cells case misidentified as respiratory system (×200); scattered lymphocytes misidentified as digestive system (×200); and acute infection inundated with neutrophile granulocytes, lymphocytes and bacteria misidentified as respiratory system (×400). **b**, Falsely classified malignant cases, from left to right: Burkitt lymphoma with scattered B lymphocytes interwoven with erythrocytes misidentified as digestive system (×400); gastric carcinoma with clusters of irregular, darker cells with crowded nuclei misidentified as respiratory system (×200); pancreatic carcinoma misidentified as respiratory system (×200); and colonic carcinoma with clusters of mucous cells adhered to each other misidentified as respiratory system (×200). **c**, Correctly classified malignant cases, from left to right: ovarian cancer, pancreatic cancer, lung cancer and ovarian cancer (×200). Smear processing by pathologists under microscope for each specimen was repeated three times independently, with the same diagnosis recorded in every instance.

For the five testing sets, on the top-1 scale, 4,765 cases were falsely classified, including 1,171 benign cases identified as malignant and 3,594 malignant cases identified as benign or other group. Of 1,171 benign cases, 261 were sorted as digestive, 352 as female reproductive, 519 as respiratory and 39 as blood and lymphatic system. Of 3,594 malignant cases, 904 were sorted as benign and 2,690 as other system. The overall false-positive rate was 11.0% (1,171 of 10,635) and the overall false-negative rate was 5.4% (904 of 16,702). We show eight common failure patterns in Fig. 5, including several characteristic cancer types. False-positive cases included one case each of reactive hyperplasia-aggregated mesothelial cells misjudged as respiratory system, of scattered lymphocytes misjudged as digestive system, of beaded degenerated histocytes misidentified as female reproductive system and of acute infection infilitrated with neutrophil granulocytes, lymphocytes and bacteria misidentified as respiratory system. In regard to the case of aggregated mesothelial cells, these are morphologically similar to well-differentiated lung adenocarcinoma with hyperchromatic nuclei. In regard to the case of beaded histocytes, bunchy degenerated histocytes resemble adenocarcinoma cells. In addition, acute inflammatory exudative hydrothorax or ascites combined with bacterial proliferation mimicking poorly differentiated carcinoma cells could be mistaken for lung adenocarcinoma. Four falsely classified malignant cases included (1) one case of gastric carcinoma with clusters of irregular, darker cells with crowded nuclei misidentified as respiratory system; (2) one case of colonic carcinoma with clusters of mucous cells adhered to each other misidentified as respiratory system; (3) one case of pancreatic carcinoma misidentified as respiratory system; and (4) one case of Burkitt lymphoma with scattered B lymphocytes interwoven with erythrocytes misidentified as digestive system. In regard to the case of pancreatic carcinoma, potential causes of AI overdiagnosis include poor smear preparation and image quality such as section folding, impurities or overstaining. Meticulous manual processing in the data-screening phase will alleviate these issues. In regard to the case of colonic carcinoma, slime occupied most of the space on the image and therefore the number of cancer cells was limited; apparently the normal structure of malignant colonic cells was disturbed by redundant excretive mucus, which may have led to the AI model overlooking this key point when making a diagnosis. Further falsely classified malignancy cases and examples of correct prediction are illustrated in Supplementary Figs. 9–11.

### Model interpretability

**Extended Data Fig. 5:**
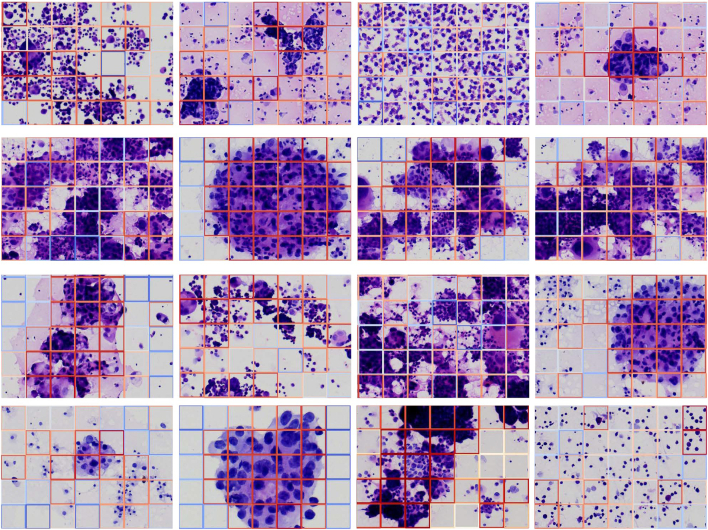
Examples of haematoxylin-eosin staining cytological attention heatmaps. The frame of each square is marked with different colors. Red frame indicates that a region is highly informative for the classification decision making and blue frame indicates that the region has lower diagnostic value. Histomorphological features contributing to prediction made by TORCH are usually featured by: organizational structures such as glandular tubules, papillary, wreath like, and compact cell clusters; cells with larger size, richer cytoplasm, obvious nuclear abnormalities, and rough, deeply stained chromatin.

The histomorphological features that contributed most to the prediction results of the AI model were identified. We used attention heatmaps to interpret model prediction results. Each image was analyzed at either 40 × 10 or 20 × 10 equivalent magnification and cut into dozens of squares, the frame of each then being marked with different colors. The algorithm calculated the correlation between each square box and tumor category. A red frame indicates that a region is highly informative for classification decision making while a blue frame indicates that the region has lower diagnostic value. In aggregate, 1,351 malignant images were randomly chosen to create corresponding attention heatmaps. The accuracy of attention heatmaps in regard to capture of the main area of isolated tumor cells was assessed by five pathologists, whose results are shown in Supplementary Table 18; the comprehensive accuracy percentage was 87.7 (95% CI 81.1–94.3%; Extended Data Table 5). Manual visual inspection showed that the histomorphological features contributing to prediction made by TORCH include (1) organizational structures such as glandular tubules; (2) papillary, wreath-like and compact cell clusters; and (3) cells of larger size with richer cytoplasm, obvious nuclear abnormalities and rough, deeply stained chromatin^23,25,42^. Examples of hematoxylin-and-eosin (H&E)-stained cytological attention heatmaps are shown in Extended Data Fig. 5 and Supplementary Figs. 12 and 13.

## Discussion

In this study we present TORCH, a deep-learning model developed to predict the primary system origin of malignant cells residing in hydrothorax and ascites. This AI model could become a valuable tool in differentiating between malignant tumor and benign disease, localization of cancer origins and aiding clinical decision making in patients with CUP. It is a challenging task to identify the origins of metastatic free tumor cells using limited clinical information and cytological images. Our model achieved robust performance across five testing sets and outstanding accuracy versus a group of four pathologists.

Improvements in pathological imaging technique, immunohistochemical methods and gene expression-profiling assays have facilitated cancer origin prediction for patients with CUP^11,28,29,31,43^. Nevertheless, the visual diagnosis of isolated tumor cells in effusion specimens by liquid-based smear remains difficult. The numbers of tumor cells in pleural effusion and ascites are usually far fewer than those in a resected tumor tissue mass. Meanwhile there is wide disparity between the original morphologic structure of the tumor specimen and tumor cell clusters, which creates major challenges for the pathologist tasked with determining differential diagnoses. From this point of view, our model could become an effective auxiliary method available to clinicopathologists. In addition, identification of tumor cells in effusion specimens is very helpful in regard to tumor staging and the selection of appropriate therapy. For example, in patients with gastric or colonic cancer the presence of free tumor cells in the abdominal cavity often indicates that the disease has progressed to a later stage^15–17^. Under this scenario, clinicians often prefer palliative therapies such as radiotherapy, chemotherapy or molecular targeted therapy; under a different scenario they could select more radical treatment methods such as surgery. TORCH was able to identify, with high accuracy, the four most common cancer systems that metastasize to hydrothorax or ascites. Compared with the group of experts, the diagnostic score for the TORCH model on an independent subset was better than that of both the senior and junior pathologists. When junior cytological pathologists with varying levels of experience were assisted by this model, their diagnostic accuracy increased significantly. Interestingly, even junior testees with little experience benefitted considerably from assistance by TORCH, with their performance approaching, but still slightly lower than, that of the senior testees. We speculated that these differences might have resulted from the level of trust invested by the pathologists in TORCH, as well as from optimal cooperation between them. The trust level of pathologists in TORCH may not have been high on the first occasion it was used and they were still relatively independent in regard to making decisions. In terms of overall accuracy and precision, the senior experts showed enhanced ability compared with their junior counterparts. One possible reason for this finding is that senior-level experts demonstrate an advanced level of meticulousness and prudence. In most pathology institutions the cytology department usually accounts for only a small part of the whole. In certain remote or undeveloped areas where cytologists are fewer in number and less experienced, our approach could be used as a reliable reference. Because pathologists do not usually base their diagnosis on H&E slices alone, our model integrates clinical data including sex, age and tissue sampling site, which is much closer to an actual clinical scenario.

We amassed a large collection of pathological images covering 32 cancer types from the The Cancer Genome Atlas (TCGA) database, and cytological images from three independent training sets, to extract image features. These large datasets guarantee applicability and generality for the development of feature extraction. We then divided the cytological images acquired from the four institutions into two main subgroups: benign and malignant cancers. In regard to malignant cases we reclassified 12 primary systems into four categories by organ system and disease type. Initially we prepared to train and validate our model directly on these 12 systems. Nevertheless, tumors from the nervous system, bone and soft tissue system and urinary system, and also melanoma and thymoma, have an extremely low incidence of metastasis to the thoracoabdominal cavity. As a result, the cytological images collected from these tumors were limited in number and insufficient for model development. Normally, in regard to ascites the digestive and female reproductive systems are the most common sources of free tumor cells; for hydrothorax the respiratory system and breast cancer are the most common sources^21–23,25^. Therefore, during network training we excluded these scarce images and included only several common systems.

In this study we selected two external cohorts for validation—one prospectively, the other retrospectively. These two fully unseen cohorts consisted of a large number of low-certainty and uncertainty cases, which represents an objective real-world cytological imaging circumstance. Following the inclusion of uncertainty cases, our AI model still demonstrated reliable capability with top-1 accuracy ranging from 70.2 to 88.1%. To further validate the performance of the TORCH model in clinical practice, we conducted a retrospective survival analysis for comparison of long-term outcomes of patients with different model predictions. Of 391 uncertainty patients with CUP, those treated in concordance with TORCH predictions demonstrated a significantly longer overall survival than patients treated in a discordant manner (27 versus 17 months, *P* = 0.006). For oncologists, under certain circumstances this offers valuable information regarding the selection of therapy. For example, among unidentified patients with CUP, mainly adenocarcinoma, around 80% of unfavorable cases were treated with empirical broad-spectrum chemotherapeutic regimens^11,28^; however, with adenocarcinoma occurring in both the digestive and female reproductive system, chemotherapeutic plans are widely divergent. To some extent our model would be a valuable auxiliary method for individual treatment schemes.

Cytological diagnosis is usually very difficult compared with that using H&E-stained sections, especially when clinical epidemiologic information is limited. Ablation studies have demonstrated the importance of synthesizing other clinical metrics during network establishment apart from merely cytological images. However, optimal use of the TORCH model in clinical practice should be implemented. In this study we used only cytological images combined with several quantifiable parameters (sex, age and specimen sampling site) for model development, without taking into account other subjective and variable factors such as medical history, site of metastasis, gene mutation, family heredity record, living habits or geographic region. For this reason, TORCH cannot be as realistic and comprehensive as the traditional method based on human experts. Future deep-learning models combining more clinically important metrics will potentially avoid pointless puncture biopsies, reduce false-positive diagnosis and decrease interobserver variability.

There are several limitations of this study. First, our model was developed based on cytological images, which means that the abundance of information extracted was not as great as whole-slide images. As a result, our model can localize tumor origins only at the organ-system level rather than identifying precise tumor origins, as done by Lu and colleagues with whole-slide images^36^. Second, our current model cannot discern other malignant disease types such as mesothelioma or those of the urinary, nervous or bone and soft tissue systems. For these rare cytological diseases, pathologists must make a comprehensive judgment based on either experience or multidisciplinary consultation. In the future we will collect further image data from the above organ systems and develop this model to further distinguish multiple broader categories. Third, patients in the four institutions are from northern, central and eastern areas of China. Although the number of cases enrolled in this study is considerable and derived from different large-scale institutions, we have not taken into account cases from other countries or other ethnic groups. Model accuracy and generalizability might be affected by variation in patients’ race and clinicians’ bias in regard to visual field selection. Fourth, although our model achieved satisfactory results, the number of images used for training remains very limited compared with computer-based visual tasks in natural image recognition^44^. In addition, the model architecture may not be optimal. We speculate that improvement could be achieved by improving the architecture of neural networks, such as taking account of the spatial association among different image patches, increasing the number of images and incorporating other data modalities such as tumor-residing area, tumor size, serum biomarkers, radiologic imaging and genetic data.

In summary, TORCH can serve as an effective tool in differentiation between malignancy and benignity, and furthermore as an auxiliary proof of concept for tumor origin prediction using cytological images. The high technical performance and potential clinical benefits of TORCH warrant further investigation in prospective randomized clinical trials.

## Methods

### Ethics and information governance

Our work received approval from the institutional review board of Tianjin Medical University Cancer Institute and Hospital (IRB no. bc2021182). Data collection and other procedures were performed in accordance with principles of Good Clinical Practice and Declaration of Helsinki guidelines (1975, revised 1983), and with other relevant ethical regulations. All patients provided written informed consent before undergoing pathological examination. Each image was anonymized before being incorporated into the framework. Likewise, only deidentified and relabeled clinical data were used for research, without the involvement of any personal patient information.

### External public datasets

For some tumors of rare origin, or those rarely metastasizing to the thoracoabdominal cavity (such as those of the nervous and bone and soft tissue systems, melanoma and head and neck tumors), the sample size of ascitic and pleural cytological smear images was limited. We acquired a large collection of pathological images from the publicly available medical dataset TCGA via the NIH Genomic Data Commons Data Portal. These data included of a wide range of tumors, both rare and common cancers, covering 32 subtypes: acute myeloid leukemia, adrenocortical carcinoma, urothelial bladder carcinoma, breast ductal carcinoma, breast lobular carcinoma, cervical cancer, cholangiocarcinoma, colorectal carcinoma, esophageal cancer, gastric adenocarcinoma, glioblastoma multiforme, head and neck squamous cell carcinoma, hepatocellular carcinoma, chromophobe renal cell carcinoma, clear cell renal cell carcinoma, papillary renal cell carcinoma, lower-grade glioma, lung adenocarcinoma, lung squamous cell carcinoma, mesothelioma, ovarian serous adenocarcinoma, pancreatic ductal adenocarcinoma, paraganglioma pheochromocytoma, prostate carcinoma, sarcoma, skin melanoma, testicular germ cell tumor, thymoma, thyroid cancer, uterine carcinosarcoma, endometrial carcinoma and uveal melanoma. In aggregate, a total of 1,360,892 image patches were clipped from whole-slide images obtained from 11,607 patients, from which the raw data amounted to approximately 20 terabytes.

### Training and testing datasets

We retrospectively collected 42,682 cases of cytological smear images from cohorts of patients who had attended three large tertiary referral institutions (Extended Data Fig. 3 and Table 1). Ultimately we enrolled 14,008 cases from Tianjin Medical University Cancer Hospital between September 2012 and November 2020, 20,820 cases from Zhengzhou University First Hospital between August 2011 and December 2020 and 7,854 cases from Suzhou University First Hospital between June 2010 and December 2020. We randomly selected 70% of these as training sets and 30% as internal testing sets. We ensured that the testing sets of patients did not overlap with those in the training set. Finally, the training sets consisted of 29,883 cases of which the three internal testing sets consisted of 12,799 cases. For ease of description we denoted these testing sets as Tianjin, Zhengzhou and Suzhou, respectively. In particular we added two independent external testing sets enrolled from Tianjin Medical University Cancer Hospital between June and October 2023 (the Tianjin-P testing set; 3,933 cases prospectively enrolled) and from Yantai Yuhuangding General Hospital between February 2013 and May 2022 (Yantai testing set; 10,605 cases retrospectively enrolled). These two external testing sets were both fully unseen cohorts that were used further to test the generalization capabilities of our model (Fig. 1).

We retrieved cytological imaging data for cells isolated from pleural and peritoneal fluid from pathologic databases. In contrast to the malignant group, the benign group consisted of patients diagnosed with benign diseases such as decompensated liver cirrhosis, nephrotic syndrome, constrictive pericarditis, pulmonary edema and pleuritis. To ensure that the diagnosis of each patient was based not only on histopathological reporting, other electronic medical records were also retrieved as ancillary verification. All pertinent clinical information—disease history, laboratory test results, family oncologic history, surgery records, endoscopic or interventional examination, chemotherapy or radiotherapy and follow-up interviews—was obtained where applicable and available. To test our model in the clinical setting scenario we divided patients into high- and low-certainty groups according to the level of supporting evidence. The high-certainty group included (1) patients whose primary tumors had been resected and with a definitive routine histopathological diagnosis and (2) patients who had undergone immunohistochemical examination by paraffin sections of cell sediment, the results of which strongly suggested the origin of malignant tumors^38,45,46^. The low-certainty group consisted of (1) patients whose primary or metastasized tumors merely underwent fine-needle puncture biopsy^47,48^ and (2) patients whose putative differential diagnosis was arrived at solely by comprehensive clinical and radiological findings. Because it is not practical to obtain a definitive ground-truth origin for some patients, with CUP, the assigned primary diagnosis of each case was reviewed by a medical team consisting of clinicians, physicians, surgeons and pathologists.

### Clinical taxonomy

To guarantee the quality of each image we asked five senior pathologists (each with >15 years experience of clinical practice) to collect corresponding pathological examination results of either sediment paraffin H&E images or surgically resected or needle biopsy specimens to verify their accuracy and authenticity. Cases were excluded for which clinical diagnosis was ambiguous or the origin of the primary tumor was unknown. A final taxonomy label was assigned to each case manually by consensus among all five pathologists. Patients treated previously by palliative chemotherapy or radiotherapy (high possibility of therapy-related changes in tumor cell morphology or high false-negative rates) were excluded from both training and testing sets. The various cancer types from these patients were first grouped into 12 subgroups according to organ function and origin. Tumors originating from esophagus, stomach, duodenum, intestine, appendix, colon and rectum were grouped under cavity digestive system; similarly, tumors from the liver, gallbladder and pancreas were grouped under secretory digestive system and those from ovary, fallopian tube, corpus uteri, cervix uterus and vagina were grouped under female genital system. Meanwhile, because of the particularity and function of the mammary gland, breast cancer was grouped under female genital system. Tumors from kidney, ureter, bladder and urethra were grouped under urinary system; to remain consistent with clinical convention, tumors from prostate, testicle and seminal vesicle were also grouped under urinary system. Tumors from lung and trachea were grouped under respiratory system. Tumors from head and neck were grouped together. Tumors from the central nervous system and peripheral nervous system were categorized as one group. Bone and soft tissue tumors were also categorized as one group. For melanoma, mesothelioma and thymoma, on account of their unique growth characteristics these were grouped individually. In addition, acute or chronic leukemia and lymphoma were grouped as blood and lymphatic system. Because some tumors (such as those of the urinary system, head and neck, nervous system, bone and soft tissue, melanoma and thymoma) rarely metastasize to the chest or abdominal serous cavity, the number of cytological images available for model training from those was limited. In the current study, specimens of mesothelioma from all four institutions were also relatively scarce. We excluded these rare cytological smear images from the above cancers and further integrated the remaining 57,220 cases into five main categories: benign, digestive system (consisting of both cavity digestive system and secretory digestive system), female reproductive system (including breast cancer), respiratory system and blood and lymphatic system (Fig. 1).

### Data curation and patching

In this study, cytology smear images rather than whole-slide images were retrieved from a real-world, clinical scenario. Initially pleural and abdominal fluids were extracted by fine-needle aspiration and directly prepared as smears for microscopic observation (JVC TK-C9501EC, Olympus BX51 at either ×400 or ×200 equivalent magnification). The pathologists selected between five and ten fields with concentrated tumor cells best representing the pathological features for semiqualitative analysis. The original image format stored in the database was 2,797 × 1,757 alike pixels. Due to variation in cell shape arising from the different tumor origins, as well as the relatively high proportion of background in cytological images, it is usually impossible to develop deep-learning models directly from these large images and thus we split each image into a list of patches of 224 × 224 pixels. We excluded blank, poorly focused and low-quality images containing severe artifacts. Extracted patches from the same image were located in a single package. For cancer-positive packages there must be at least one patch that includes tumor cells; for negative packages, no patch must contain tumor cells.

### Development of the feature extractor

We used self-supervised feature representation learning with momentum contrast (MoCo) as learning representation for histological and cytological images. The key concept of this method is to minimize the contrastive loss of different augmented versions of a given image. The feature extractor is a 50-layer residual neural network (ResNet) consisting of four residual blocks, followed by a multilayer perceptron (MLP) to project the outputs from ResNet into a latent space where contrastive learning is performed. The use of MLP has proved to be beneficial in regard to contrastive learning. The framework of MoCo is a Siamese network consisting of two feature encoders whose parameters are denoted as *θ*_*k*_ and *θ*_*q*_. MoCo learns similar/dissimilar representations from images that are organized into similar/dissimilar pairs, which can be formulated as a dictionary lookup problem. For a given image *x* we perform random data augmentation for *x* giving rise to *x*_*k*_ and *x*_*q*_; *x*_*k*_ is fed into *θ*_*k*_ and *x*_*q*_ into *θ*_*q*_. This problem can be optimized efficiently by InfoNCE loss^49^:

$${{\mathcal{L}}}_{q,{k}^{+},\left\{{k}^{-}\right\}}=-\log \frac{\exp (q.{k}^{+}/\tau )}{\exp \left(q.\frac{{k}^{+}}{\tau }\right)+\sum _{{k}^{-}}\exp\left(q.\frac{{k}^{-}}{\tau }\right)}$$

where *q* is a query representation and *k*^+^ is the representation of a similar key sample of *q*, both of which are obtained via data augmentation for the same image. {*k*^−^} is the set of representation of dissimilar samples of *q*, which are obtained via data augmentation for the other images. The size of dissimilar samples was set to 65,536. The two feature encoders, *θ*_*k*_ and *θ*_*q*_, are updated in different ways, whereas *θ*_*q*_ is updated by back-propagation and *θ*_*k*_ is updated according to *θ*_*k*_ ← *mθ*_*k*_ + (1 − *m*)*θ*_*q*_. *m* ∈ (0, 1) is the momentum coefficient and was set to *m* = 0.999 in our study. Hyperparameter *τ* was set to 0.07. We used stochastic gradient descent to train the network for 200 epochs with an initial learning rate of 0.015, weight decay of 1 × 10^−4^ and batch size of 128 on four graphics processing units. The learning rate was scheduled by cosine decaying. Specifically, the learning rate at the *i*th epoch was set to initial_lr × 0.5 × (1.0 + cos(π × *i*/*n*)) where *n* is the total number of training epochs, set to 200 in this study. The ResNet encoder is eventually used as feature extractor. Data augmentation includes random resize and crop, color jittering, grayscaling, Gaussian blurring, flipping and subsequently normalization by the mean and standard deviation of channels R, G and B. In total, 1,360,892 histological image patches from TCGA and 29,883 cytological image patches were used for the development of the histological feature extractor and cytological feature extractor, respectively. We eventually obtained two feature extractors: cytological and histological feature extractors.

For a given cytological image with *n* tiling patches we converted each patch into a feature vector of 1,024 dimensions. These feature vectors were then combined as feature matrix *X*_image_ of *n* rows and 1,024 columns. Besides image features we took clinical parameters as inputs including age, sex and specimen sampling site. In this scenario we embedded age, sex and specimen sampling site into a vector of 1,024 dimensions, denotated as *X*_age_, *X*_sex_ and *X*_location_. The input to the attention-based MIL classifier can be set to *X* = *X*_image_ and *X* = *X*_image_ + *X*_age_ + *X*_sex_ + *X*_location_.

### Model training

Because each extracted patch represents only a small fraction of tumor features or tissue content, labeling these with patient-level diagnosis is inappropriate. We therefore used a weakly supervised machine learning method and trained a multitask neural network model named TORCH while taking into account information from the entire package. Parameters including sex, age and specimen sampling site (hydrothorax and ascites), combined with cytological images, were taken as inputs. We trained our model in an end-to-end fashion with stochastic gradient descent for 100 epochs at a constant learning rate of 2 × 10^−4^, weight decay of 1 × 10^−5^ and batch size of 1 using the Adam optimizer^50^. From epoch 60 and beyond, the model with the lowest validation loss was selected as the optimal model. We trained four deep neural networks individually on the training set. These networks included attention-based, multiple-instance learning (AbMIL), AbMIL with multiple attention branches (AbMIL–MB), transformer-based MIL (TransMIL) and TransMIL with cross-modality attention. These methods can be categorized as either attention- or transformer-based MILs. The objectives and differences of these four algorithms are shown in Supplementary Table 19. Image features were extracted using the cytological and histological feature extractor. For each network we trained and obtained three models for different combination of inputs: (1) cytological image features plus age, sex and specimen sampling sites; (2) histological image features plus age, sex and specimen sampling sites; and (3) cytological and histological image features plus age, sex and specimen sampling sites. As a result, we obtained 12 trained models. Finally we performed model ensembling by averaging the prediction probabilities from these models. Model training and evalutation were performed with PyTorch (v.1.12.1) on a DGX A100 computing server.

### AbMIL

In the setting of multiple-instance learning, a cytological image is considered as a bag and image patches from that cytological image are instances^51,52^. For a cytological image with *k* patches we can obtain a feature matrix, denoted as [*x*_1_, *x*_2_, …, *x*_*k*_]^*T*^; *x*_*i*_ is the feature vector of the *i*th image patch output from the feature extractor. A two-layer, fully connected neural network transforms *x*_*i*_ into latent vector *h*_*i*_:

$${h}_{i}={{\mathrm{ReLU}}}\left({W}_{2}\right.\left({{\mathrm{ReLU}}}\left({W}_{1}{x}_{i}+{b}_{1}\right)\right)+{b}_{2}$$

where *W*_1_, *W*_2_, *b*_1_ and *b*_2_ are parameters and ReLU is the activation function. The attention weight *a*_*i*_ for *h*_*i*_ is defined as^51^

$${a}_{i}=\frac{\exp (\tanh (V{h}_{i})\odot {{\mathrm{sigmoid}}}(U{h}_{i}))}{{\sum }_{j=1}^{k}\exp (\tanh (V{h}_{j})\odot {{\mathrm{sigmoid}}}(U{h}_{j}))}$$

where *V* and *U* are weight parameters and tanh and sigmoid are activation functions. Attention pooling was applied to obtain the sample-level features:

$$\begin{array}{ll}{Z}={{H}^{T}}{A}\\{\rm{where}}\;{A}=\{{{a}_{1}},{{a}_{2}},\ldots,{{a}_{{k}}}\}\;{\rm{and}}\;H=\{{{h}_{1}},{{h}_{2}},\ldots,{{h}_{k}}\}.\end{array}$$

Subsequently, a fully connected layer parameterized as *W*_3_ and *b*_3_, followed by sofmtax, was used to transform sample-level features into probabilities:

$$p={{\mathrm{softmax}}}\left({W}_{3}Z+{b}_{3}\right).$$

### AbMIL–MB

This approach is an extension of attention-based deep MIL based on Lu et al.^52^. Let ${{\mathbf{z}}}_{k}\in {{\mathbb{R}}}^{2,048}$ denote the patch-level representation extracted from the feature extractor. A fully connected layer, ${W}_{1}\in {{\mathbb{R}}}^{512 \times 2,048}$, projects **z**_*k*_ into a 512-dimensional vector **h**_*k*_ = *W*_1_**z**_*k*_. Suppose the attention network consists of two layers. ${U}_{a}\in {{\mathbb{R}}}^{384 \times 512}$ and ${V}_{a}\in {{\mathbb{R}}}^{384 \times 512}$; subsequently the attention network splits into *N* parallel attention branches, ${W}_{a,1},{W}_{a,2},\ldots,{W}_{a,N}\in {{\mathbb{R}}}^{1 \times 384}$. Then, *N* parallel classifiers (that is, ${W}_{c,1},{W}_{c,2},\ldots, {W}_{c,N}\in {{\mathbb{R}}}^{1 \times 512}$) are built to create a class-specific prediction for each cytological image. The attention score of the *k*th patch for the *i*th class *a*_*k*,*i*_ is calculated as

$${a}_{i,k}=\frac{\exp \{{W}_{a,i}(\tanh \left({V}_{a}{{{{\mathbf{h}}}}}_{k}\right)\odot {{\mathrm{sigmoid}}}\left({U}_{a}{{{{\mathbf{h}}}}}_{k}\right))\}}{{\sum }_{j=1}^{K}\exp \{{W}_{a,i}(\tanh \left({V}_{a}{{\mathbf{h}}}_{j}\right)\odot {{\mathrm{sigmoid}}}\left({U}_{a}{{\mathbf{h}}}_{j}\right))\}}.$$

The aggregated representation for a cytological image for the *i*th class is given by

$$\displaystyle{{\mathbf{h}}}_{{{\mathrm{cyto}}},i}=\displaystyle\mathop{\sum }\limits_{k=1}^{K}{a}_{i,k}{{\mathbf{h}}}_{k}.$$

The logit value for a cytological image is calculated as

$${s}_{{{\mathrm{cyto}}},i}={W}_{c,i}{{\mathbf{h}}}_{{{\mathrm{cyto}}},i}.$$

Softmax function is applied to convert *s*_cyto,*i*_ into the predicted probability distribution over each class.

### TransMIL

TransMIL for whole-slide image classification was investigated in our recent study^53^ and in a study by Wagner et al.^54^. For a given cytological image we first split it into multiple 224 × 224 image patches. Let ${z}_{k}\in {{\mathbb{R}}}^{2,048}$ denote the patch-level representation. A fully connected layer ${W}_{1}\in {{\mathbb{R}}}^{384 \times 2,048}$ projects *z*_*k*_ into a 384-dimensional vector **h**_*k*_ = *W*_1_**z**_*k*_. Clinical features including sex, age and sample origin (that is, ascites or pleural effusion) are independently embedded into a 384-dimensional vector: *h*_sex_, *h*_age_ and *h*_origin_. Similar to the vision transformer, we prepend a learnable embedding *h*_class_ to the sequence of image patches. The state of *h*_class_ at the output of the transformer encoder is used as the representation of that cytological image. We then concatenate the patch-level features with clinical features as *h* = {*h*_class_, *h*_1_, *h*_2_, …, *h*_*k*_, *h*_sex_, *h*_age_, *h*_origin_}. The position embeddings $p\in {{\mathbb{R}}}^{(k+4) \times 384}$ are added to *h* to retain positional information, giving rise to input *x* = *h* + *p.*

The concatenated features $x\in {{\mathbb{R}}}^{(k+4) \times 384}$ are passed through the transformer encoder, which consists of three layers, to make a diagnostic prediction. The transformer encoder layer comprises a multiheaded self-attention and a positionwise, feedforward neural network (FFN). The *i*th self-attention head is formulated as

$${{\mathrm{Attentio}{n}}}_{i}\left({Q}_{i},{K}_{i},{V}_{i}\right)={{\mathrm{softmax}}}\left(\frac{{Q}_{i}{K}_{i}^{T}}{\sqrt{{d}_{k}}}\right){V}_{i}$$

where *Q*_*i*_, *K*_*i*_ and *V*_*i*_ are three matrices that are linearly projected from the concatenated feature matrix *x* and *d*_*k*_ is the dimension of *Q*_*i*_, which is used as scaling factor. In this study *d*_*k*_ is set to 64. *Q*_*i*_, *K*_*i*_, *V*_*i*_ = LP(*x*), where LP represents linear projection. Multiheaded self-attention is the concatenation of different self-attention heads:

$${{\mathrm{MultiHeadAttention}}}\left(Q,K,V\right)={{\mathrm{Concat}}}\left({{\mathrm{Attention}}}_{1},\ldots ,{{\mathrm{Attention}}}_{h}\right){W}^{o}$$

where *W*^*o*^ represents the learnable projection matrix. The pointwise FFN has two linear layers with ReLU activation between:

$${{\mathrm{FFN}}}\left(x\right)=\max \left(0,x{W}_{1}+{b}_{1}\right){W}_{2}+{b}_{2}$$

where *W*_1_ and *W*_2_ are weights and *b*_1_ and *b*_2_ are bias. Layerwise normalization is applied in the front and rear of FFN, and residual connection is employed to improve information flow. The representation of the learnable classification vector obtained from the last transformer encoder layer is passed through a linear classifier to make a diagnostic prediction.

### TransMIL with cross-modality attention

TransMIL simply uses concatenation for multimodal data fusion but does not exploit interconnections between different data modalities. Zhou and colleagues proposed a state-of-the-art, transformer-based representation learning model capable of exploiting intermodality between image and clinical features for clinical diagnosis^55^. These authors also proposed a multimodal attention block capable of learning fused representations by capturing interconnections among tokens from the same modality or across different modalities, and subsequently using self-attention blocks to learn holistic multimodal representations. A classification head is then added to produce classification logits. For the convenience of description, let ${{\boldsymbol{z}}}_{k}\in {{\mathbb{R}}}^{2,048}$ denote patch-level representation. A fully connected layer ${W}_{1}\in {{\mathbb{R}}}^{384 \times 2,048}$ projects **z**_*k*_ into a 384-dimensional vector **h**_*k*_ = *W*_1_**z**_*k*_. Similar to the vision transformer, we prepend a learnable embedding **h**_class_ to the sequence of image patches. Therefore, a cytological image split into *N* image patches is represented by **h** = {**h**_class_, **h**_1_, **h**_2_, …, **h**_*k*_}. The position embeddings ${\mathbf{p}}\in {{\mathbb{R}}}^{(k+1)\times 384}$ are added to **h** to retain positional information, giving rise to input

$${{\mathbf{x}}}_{{\mathbf{I}}}={\mathbf{h}}+{\mathbf{p}}.$$

Clinical features including sex, age and sample origin (that is, ascites or pleural effusion) are independently embedded into a 384-dimensional vector, **h**_sex_, **h**_age_ and **h**_origin_, and subsequently concatenated to produce a sequence of clinical features, **x**_c_ = {**h**_sex_, **h**_age_, **h**_origin_}. We used three transformer encoder layers, the first two being stacked multimodal attention blocks while the third was a self-attention block according to the original study.

Suppose the LP of **x**_*I*_ and **x**_c_ produces

$${{\mathbf{Q}}}_{{\mathbf{I}}},{{\mathbf{K}}}_{{\mathbf{I}}},{{\mathbf{V}}}_{{\mathbf{I}}}={\mathrm{LP}}({{\mathbf{x}}}_{{\mathbf{I}}})$$

and

$${{\mathbf{Q}}}_{{\mathbf{C}}},{{\mathbf{K}}}_{{\mathbf{C}}},{{\mathbf{V}}}_{{\mathbf{C}}}={\mathrm{LP}}({{\mathbf{x}}}_{{\mathbf{C}}}).$$

The operations of multimodal attention block at the *i*th layer can then be summarized as

$${\mathbf{\mathcal{X}}}_{{\mathbf{I}}}^{l}={{\mathrm{Attention}}}\left({Q}_{I},{K}_{I},{V}_{I}\right)+{{\mathrm{Attention}}}({Q}_{I},{K}_{T},{V}_{T})$$

and

$${\boldsymbol{\mathcal{X}}}_{{\mathbf{C}}}^{l}={{\mathrm{Attention}}}\left({Q}_{C},{K}_{C},{V}_{C}\right)+{{\mathrm{Attention}}}({Q}_{C},{K}_{I},{V}_{I})$$

whereas

$${{\mathrm{Attention}}}\left(Q,K,V\right)={{\mathrm{softmax}}}\left(\frac{Q{K}^{T}}{\sqrt{{d}_{k}}}\right)V.$$

Next, ${{\mathcal{X}}}_{{\mathbf{I}}}^{l}$ and ${{\mathcal{X}}}_{{\mathbf{C}}}^{l}$ are passed through a layer-normalization (LayerNorm) layer and an MLP and subsequently with residual connection to the input:

$${{\mathcal{X}}}_{{\mathbf{I}}}^{l+1}={{\mathrm{MLP}}}\left({{\mathrm{LayerNorm}}}\left({{\mathcal{X}}}_{{\mathbf{I}}}^{l}\right)\right)+{{\mathcal{X}}}_{{\mathbf{I}}}^{l}$$

and

$${{\mathcal{X}}}_{{\mathbf{C}}}^{l+1}={{\mathrm{MLP}}}\left({{\mathrm{LayerNorm}}}\left({{\mathcal{X}}}_{{\mathbf{C}}}^{l}\right)\right)+{{\mathcal{X}}}_{{\mathbf{C}}}^{l}.$$

Next, ${{\mathcal{X}}}_{{\mathbf{I}}}^{l+1}$ and ${{\mathcal{X}}}_{{\mathbf{C}}}^{l+1}$ are passed through the following multimodal attention layer, producing new representation outputs ${{\mathcal{X}}}_{{\mathbf{I}}}^{l+2}$ and ${{\mathcal{X}}}_{{\mathbf{C}}}^{l+2}$. ${{\mathcal{X}}}_{{\mathbf{I}}}^{l+2}$ and ${{\mathcal{X}}}_{{\mathbf{C}}}^{l+2}$ are then concatenated and passed through a standard transformer encoder block. Multiple attention heads are allocated for both multimodal attention and self-attention blocks. For classification purposes, average pooling is performed for representations from the standard transformer encoder block. This average representation is passed through a classification head, consisting of a two-layer MLP, to produce the final classification logits.

### Interpretability and visualization

For an input image we can directly obtain the attention scores for each image patch on that image when it is passed through the trained TORCH model^51,52^. For a cytological image with *k* patches, the attention score for the *i*th image patch calculated in the model is given by

$${a}_{i}=\frac{\exp (\tanh (V{h}_{i})\odot {{\mathrm{sigmoid}}}(U{h}_{i}))}{\mathop{\sum }\nolimits_{j=1}^{k}\exp (\tanh (V{h}_{j})\odot {{\mathrm{sigmoid}}}(U{h}_{j}))}$$

where *V* and *U* are weight parameters, tanh and sigmoid are activation functions and *h*_*i*_ is the representation feature of the *i*th image patch. Therefore, the attention scores for image patches in that cytological image are represented as *A* = [*a*_1_, *a*_2_, …, *a*_*k*_]. The attention score of each image patch represents the association of that patch on the classification output, thereby providing an intuitive interpretation. The interpretability heatmap is created by overlaying attention scores *A* onto the original cytological image. Specifically, we overlaid square boxes of different colors, as represented by the attention scores following the color scheme coolwarm implemented in the matplotlib python package, onto the original cytological image. A reddish color indicates a stronger association of that image patch on the classification, while a bluish color indicates a weaker association.

### AI architecture evaluation by different classifications

#### Cancer-positive versus cancer-negative classification

Given a cytological image, TORCH outputs the five probabilities as either digestive system (*P*_digestive_), female reproductive system (*P*_female_), respiratory system (*P*_respiratory_), blood and lymphatic system (*P*_blood-lymph_) or benign group (*P*_benign_). The cancer-positive probability is calculated as *P*_cancer_ = 1 − *P*_benign_. Together with the true label, we can use *P*_cancer_ to measure the accuracy, sensitivity, specificity and positive and negative predictive values of our model in identification of cancer-positive cases.

#### Classification of primary tumor origin

If a case is identified as malignant, it will be predicted as one of following four groups according to the highest predicted probability: digestive system, female reproductive system, respiratory system and blood or lymphatic system. For each testing set, the microaveraged one-versus-rest ROC curve was used to demonstrate the overall multiclassification performance of our model. In addition to the metrics mentioned above, we used top-*n* accuracy to evaluate the performance of origin prediction as reported by Lu and colleagues^36^. In the present study we set *n* as 1, 2 and 3. Top-1, -2 and -3 accuracy was used to measure frequency in regard to the correct label found, and to make the maximum confidence prediction. Top-*n* accuracy looks at the *n*th classes with the highest predicted probabilities when calculating accuracy. If one of the top-*n* classes matches the ground-truth label, the prediction is considered to be accurate.

#### Classification stratified by specimen sampling site

There is a tendency for malignant tumors to metastasize to the thoracoabdominal cavity. The incidence of metastasis to hydrothorax or ascites varies by tumor origin. Both lung and breast cancer are prone to thoracic metastasis, while gastrointestinal tumors are more likely to metastasize to the abdominal cavity. To confirm the variation in model performance between pleural effusion and ascites, we divided cytology smears into hydrothorax and ascites groups, respectively, and evaluated our model on each group. For the five testing sets, 16,892 thoracic cytology smear image cases and 10,445 abdominal cytology smear image cases were enrolled.

#### Classification stratified by carcinoma versus noncarcinoma

Carcinoma and noncarcinoma are two main types of malignant tumor, but with different origins. Carcinoma originates from epithelial tissue, with tumor cells arranged in nests and distinct parenchymal and stromal boundaries. In this study, in regard to those four main categories, noncarcinomatous tumors include those originating from mesenchymal tissue, malignant teratoma and the blood and lymphatic system. Sarcoma originates from mesenchymal tissue (mesoblastema) with its tumor cells scattered and interwoven between both parenchyma and stroma. We therefore divided test cases into carcinoma and noncarcinoma groups for separate assessment of the efficacy of our model on each group.

#### Classification stratified by adenocarcinoma versus nonadenocarcinoma

On cytological smears, metastatic adenocarcinoma cells are typically arranged in a three-dimensional mode with a glandular mass, more mucus in the cell cytoplasm and obvious nucleoli. Given this, and based on the morphology and characteristics of scattered tumor cells, for some typical tumors pathologists can visually distinguish between adenocarcinoma and squamous cell carcinoma. However, in the absence of routine histopathological whole-slide and immunohistochemical results, it is difficult to identify the origins of these cells according to their macroscopic appearance alone. To further evaluate the efficacy of our model in regard to different pathological subtypes, we grouped carcinomata from testing sets roughly into adenocarcinoma and nonadenocarcinoma groups and evaluated our model on each group separately. The nonadenocarcinoma group included mainly squamous cell carcinoma, sarcomatoid carcinoma, adenosquamous carcinoma, papillary carcinoma, large cell carcinoma, small cell carcinoma, transitional epithelial carcinoma, basal cell carcinoma and undifferentiated carcinoma. In this study the adenocarcinoma subset included mainly hepatopancreatobiliary, gastrointestinal, lung, breast and female genital (ovary and corpus uteri) tumors. The squamous cell carcinoma subset included mainly pulmonary, esophageal and female genital (cervix uterus and vagina) tumors.

#### Evaluation on real-world data

To verify the generalization of our model in real-world settings, we included two fully unseen external testing sets, Tianjin-P and Yantai. We prospectively enrolled 4,520 consecutive cases from 20 June to 5 October 2023 at Tianjin Cancer Hospital as the Tianjin-P testing set. These cases were obtained from outpatient or inpatient departments and had not been manually abridged. Of these 4,520 cases, 1,881 were putatively diagnosed by comprehensive clinical and radiological findings and classified as low-certainty cases; the origin of 587 cases could not be determined clinically, and these were then classified as uncertainty CUP patients. The Yantai testing set consisted of 12,467 cases retrospectively enrolled from Yantai Hospital between February 2013 and May 2022. Of these 12,467 cases, 4,646 were classified as low certainty and 1,862 as uncertainty. Because data on the performance of our model on uncertainty cases are not available due to the absence of true labels for these cases, we assessed performance on cases with known cancer origins (3,933 cases from Tianjin-P and 10,605 from Yantai). The upper-bound accuracy of our model can be estimated by assuming that our model achieves 100% accuracy in prediction of cancer origins for all uncertainty cases, whereas lower-bound accuracy can be estimated by assuming that it achieves 0% accuracy for uncertainty cases.

### AI versus pathologists

To compare the performance of TORCH with that of experienced practicing pathologists, we randomly selected 495 cytological images from three internal testing sets for manual interpretation. Four practicing pathologists (two senior experts: X.J.J. and W.N., mean 16 years of clinical experience; and two junior experts: F.J.J. and H.J.Y., mean 5 years of clinical experience) were presented with an entire clinicopathological dataset (sex, age, specimen sampling site) of every selected smear image case. Every pathologist checked all 495 selected cases. We used the following scoring scheme^36^ to quantify and compare the performance of our model with these four pathologists. For a given case we assign a diagnostic score *η* based on the prediction:

*η* = 0 if benign disease is misclassified as malignant tumor or vice versa;

*η* = 1 if tumor origin is misclassified; and

*η* = 2 if prediction is correct.

We therefore obtained two scoring vectors: ${a}_{{{\mathrm{TORCH}}}}=\{{\eta }_{1}^{{\prime} },{\eta }_{2}^{{\prime} },\ldots ,{\eta }_{495}^{{\prime} }\}$ for TORCH and ${a}_{{{\mathrm{pathologist}}}}=\{{\eta }_{1}^{* },{\eta }_{2}^{* },\ldots ,{\eta }_{495}^{* }\}$ for each pathologist. Statistical comparison was conducted to assess variation betweeen TORCH and pathologists and between pathologists with and without assistance from TORCH.

To investigate whether the junior pathologists’ diagnostic ability could be improved with the assistance of TORCH, we randomly selected 496 additional cases (not overlapping with the previous 495 cases) from three internal testing sets and present the prediction results from TORCH for these two pathologists as reference. They were asked to carry out differential diagnosis independently, with freedom to choose whether they trusted AI. We then compared their diagnostic scores to measure whether assistance by TORCH could improve junior pathologists’ diagnostic ability.

### Ablation experiment

To assess the benefit of incorporating clinical variables as inputs in addition to cytology smear images, we conducted ablation experiments by exclusion of epidemiological data from prediction of tumor origin^36^. We trained the model solely on cytology smear images by exclusion of clinical variables including sex, age and specimen sampling site. We then compared the performance of the ablation model trained using cytology smear images as the only input with that of the TORCH model trained using both cytology smear images and the above three parameters.

To explore the relationship between clinical variables and cytological images, we perturbed each clinical variable for the model-trained clinical variables and subsequently assessed differences with respect to its differences. For the ease of description, let *x*, *a*, *s* and *t* denote image features, age, sex and specimen sampling site, respectively, and therefore the input to TORCH (denoted as *f*) is represented as *X* = {*x*, *a*, *s*, *t*}. To assess the impact of the relationships between age and cytological image on model performance, we randomly replaced the age value with a random number sampled from the range 18–90, giving rise to *X*^age^ = {*x*, *a*′, *s*, *t*}. To assess the impact of the relationships between sex and cytological image on model performance, we reversed the sex value for a given patient, replacing male with female if that patient was male and vice versa. In this way we obtained a new data point representing the perturbed sampling site of sex *X*^sex^ = {*x*, *a*, *s*′, *t*}. In a similar manner, to assess the impact on model performance of the relationships between specimen sampling site and cytological image, we reversed the specimen sampling site giving rise to a new data point representing perturbed sampling site *X*^site^ = {*x*, *a*, *s*, *t*′}. Suppose the top-1 accuracy is calculated according to function ∅, the top-1 accuracies of *X*, *X*^age^, *X*^sex^ and *X*^site^ are represented as

$$\tau ={{\varnothing }}(\;f\left(X\right)),$$

$${\tau }^{{{\mathrm{age}}}}={{\varnothing }}(\;f\left({X}^{{{\mathrm{age}}}}\right)),$$

$${\tau }^{{{\mathrm{sex}}}}={{\varnothing }}(\;f\left({X}^{{{\mathrm{sex}}}}\right))$$

and

$${\tau }^{{{\mathrm{site}}}}={{\varnothing }}(\;f({X}^{{{\mathrm{site}}}})),$$

respectively.

Therefore, the impact of age, sex and specimen sampling site in relation to cytological image on model performance can be measured as

$${\Delta }^{{{\mathrm{age}}}}=\left(\,\tau -{\tau }^{{{\mathrm{age}}}}\right)/\tau,$$

$${\Delta }^{{{\mathrm{sex}}}}=\left(\,\tau -{\tau }^{{{\mathrm{sex}}}}\right)/\tau$$

and

$${\Delta }^{{{\mathrm{site}}}}=\left(\,\tau -{\tau }^{{{\mathrm{site}}}}\right)/\tau,$$

respectively.

### Clinical treatment and TORCH prediction

To investigate whether our TORCH model could assist oncologists in tracing the cancer origin of patients with CUP and provide benefit for subsequent treatment, we retrospectively collected 762 uncertainty cases treated at Tianjin Medical University Cancer Hospital between April 2020 and February 2023. All patients had received individualized treatment following detection of pleural and peritoneal serous effusions. These patients underwent comprehensive clinical imaging examination on admission, but their primary tumor origins could still not be identified. Following screening, 87 patients with incomplete hospitalized therapy data and 284 with missing follow-up information were excluded. Eventually we enrolled a cohort of 391 patients with CUP defined as uncertainty cases, of which 310 received palliative chemotherapy and targeted drugs combined with or without radiotherapy. The remaining 81 patients received surgery or supportive treatment due to various contraindications to chemotherapy. During hospitalization, all clinical data of these patients were collected, including differential diagnosis for possible primary cancer origin, biopsy site, initial chemotherapy, tumor-targeted monoclonal antibody therapy and intensity-modulated radiation therapy plans. We then asked three senior oncologists (mean 15 years of experience) to review these clinical data and determine whether TORCH-predicted tumor origins were concordant or discordant with the initial firstline treatment plan. Due to the fact that the majority of these 310 cases were patients with late-stage cancer involving multiple organ metastases, and that drug resistance occurred frequently, we referred the initial firstline palliative chemotherapy plan as the main evaluation benchmark. Response Evaluation Criteria in Solid Tumors was used as the standard reference for treatment effect assessment. Karnofsky score was applied as function status scoring criteria, with scoring by oncologists before and after chemotherapy, respectively. Overall survival was calculated as the time interval from the date of admission to either that of death (due to either cancer cachexia or any other cause) or the follow-up date (27 September 2023). According to whether TORCH-predicted tumor origins were concordant with treatment plans, we divided these 391 patients into the concordant and discordant groups. The former and latter included patients who had received treatment plans that were concordant or discordant, respectively, with TORCH-predicted tumor origins. The three senior clinical oncologists made comprehensive judgments (concordant or discordant) according to National Comprehensive Cancer Network guidelines^56^, standard Chinese expert consensus^57^, patients’ hospitalization records and their own clinical experience. They were blind to follow-up information when making judgments.

### Assessment of inter-rater agreement rate among pathologists

We calculated the inter-rater agreement rate for the four pathologists involved in manual interpretation of cytological images. We used Fleiss’ kappa (*κ*)^58,59^ to measure inter-rater reliability when including multiple raters and more than two categories, which was calculated according to

$$\kappa =\frac{{p}_{\mathrm{o}}-{p}_{\mathrm{e}}}{1-{p}_{\mathrm{e}}}$$

where *p*_o_ is the observed agreement rate and *p*_e_ the expected agreement rate. According to Landis and Koch^41^, interpretation of *κ* was grouped into six agreement categories: poor (*κ* < 0), slight (0 ≤ *κ* < 0.2), fair (0.21 ≤ *κ* < 0.4), moderate (0.41 ≤ *κ* < 0.60), substantial (0.61 ≤ *κ* < 0.80) and almost perfect (0.81 ≤ *κ* ≤ 1.0).

### Statistics

Area under the receiver operating characteristic curve was used as the primary metric to measure classification performance. Confidence intervals of AUROC were computed using DeLong’s method implemented in the R package pROC (v.1.17.0.1). The Clopper–Pearson method^60^ was used to calculate accuracy, sensitivity, specificity and positive predictive and negative predictive values. We conducted permutation testing to determine any statistical difference across the five categories in terms of AUROC, precision and recall rate. Fleiss’ kappa was used to measure inter-rater agreement among pathologists (R package irr, v.0.84). Rates of mortality were censored in September 2023 and calculated using the Kaplan–Meier method. The log-rank test was employed to test for differences between Kaplan–Meier survival curves. Statistical analysis was performed with R software (v.3.9.1), pROC (v.1.17.0.1) and sklearn (v.0.24.1).

### Reporting summary

Further information on research design is available in the Nature Portfolio Reporting Summary linked to this article.

## Online content

Any methods, additional references, Nature Portfolio reporting summaries, source data, extended data, supplementary information, acknowledgements, peer review information; details of author contributions and competing interests; and statements of data and code availability are available at 10.1038/s41591-024-02915-w.

### Supplementary information

Supplementary Information Supplementary Figs. 1–13 and Tables 5 and 19.

Reporting Summary

Supplementary Data Supplementary Tables 1–4 and 6–18.

## Figures and Tables

**Extended Data Table 1:**
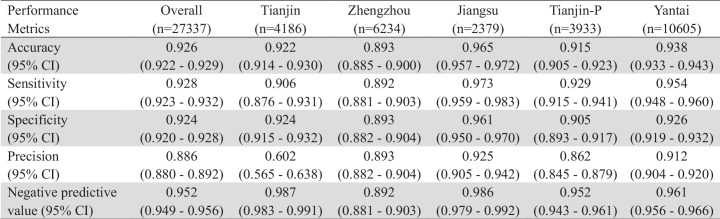
Classification performance of TORCH model on malignant versus benign diseases

Classification performance of TORCH model on malignant versus benign diseases.

**Extended Data Table 2:**
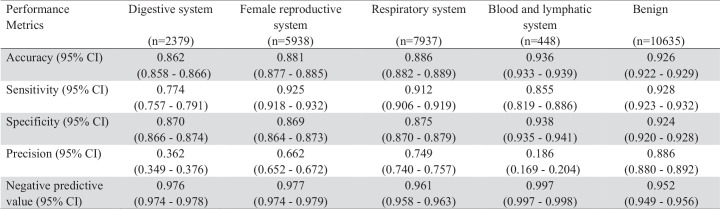
Classification performance of TORCH model on five categories

Classification performance of TORCH model on five categories.

**Extended Data Table 3:**
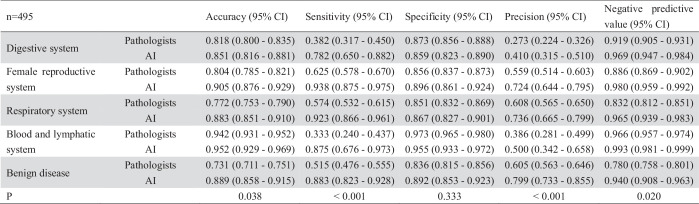
Classification performance of pathologists versus TORCH model

Classification performance of pathologists versus TORCH model.

**Extended Data Table 4:**
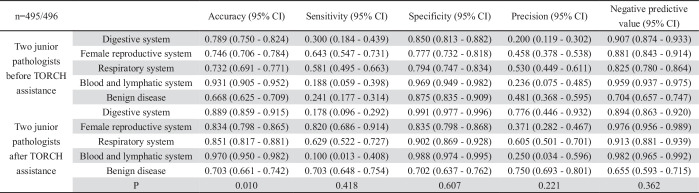
Classification performance of junior pathologists with and without TORCH assistance

Classification performance of junior pathologists with and without TORCH assistance.

**Extended Data Table 5:**
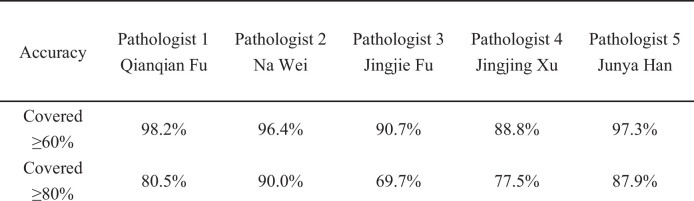
The accuracy of attention heatmaps capturing main area of tumor cells assessed by pathologists

The accuracy of attention heatmaps capturing main area of tumor cells assessed by pathologists.

## Data Availability

TCGA whole-slide image data are available from NIH genomic data commons (https://portal.gdc.cancer.gov). The supporting data generated in this study are provided in Supplementary Information. Sample data and cytological images for communication are given at figshare via 10.6084/m9.figshare.25270066 (ref. ^61^). The full treatment plan, survival information and other deidentified clinical data used in treatment concordance analysis are available in Supplementary Table 17. Restrictions apply to the availability of cytological image data, which were used with institutional permission through IRB approval for the current study and are thus not publicly available. Please email any request for academic use of cytological imaging data to either the corresponding author (lixiangchun@tmu.edu.cn) or first author (tianfei@tmu.edu.cn). All requests will be evaluated based on institutional and departmental policies to determine whether the data requested are subject to intellectual property or patient privacy obligations. Data can be shared for noncommercial academic purposes only and will require a formal material transfer agreement. Requests will be processed within 3 weeks.

## Code availability

Source code for model development is publicly available at GitHub via https://github.com/deeplearningplus/TORCH (ref. ^62^).
