# Supplementary material for: Prediction of tumor origin in cancers of unknown primary origin with cytology-based deep learning
Source: Nat Med. 2024 Apr 16;30(5):1309–19. doi: 10.1038/s41591-024-02915-w (PMC11108774; doi:10.1038/s41591-024-02915-w)
Supplement: Supplementary file 1 — Supplementary Figs. 1–13 and Tables 5 and 19. [file 41591_2024_2915_MOESM1_ESM.pdf]

nature medicine

Article https://doi.org/10.1038/s41591-024-02915-w

Prediction of tumor origin in cancers of unknown primary origin with cytology-based deep learning

In the format provided by the authors and unedited

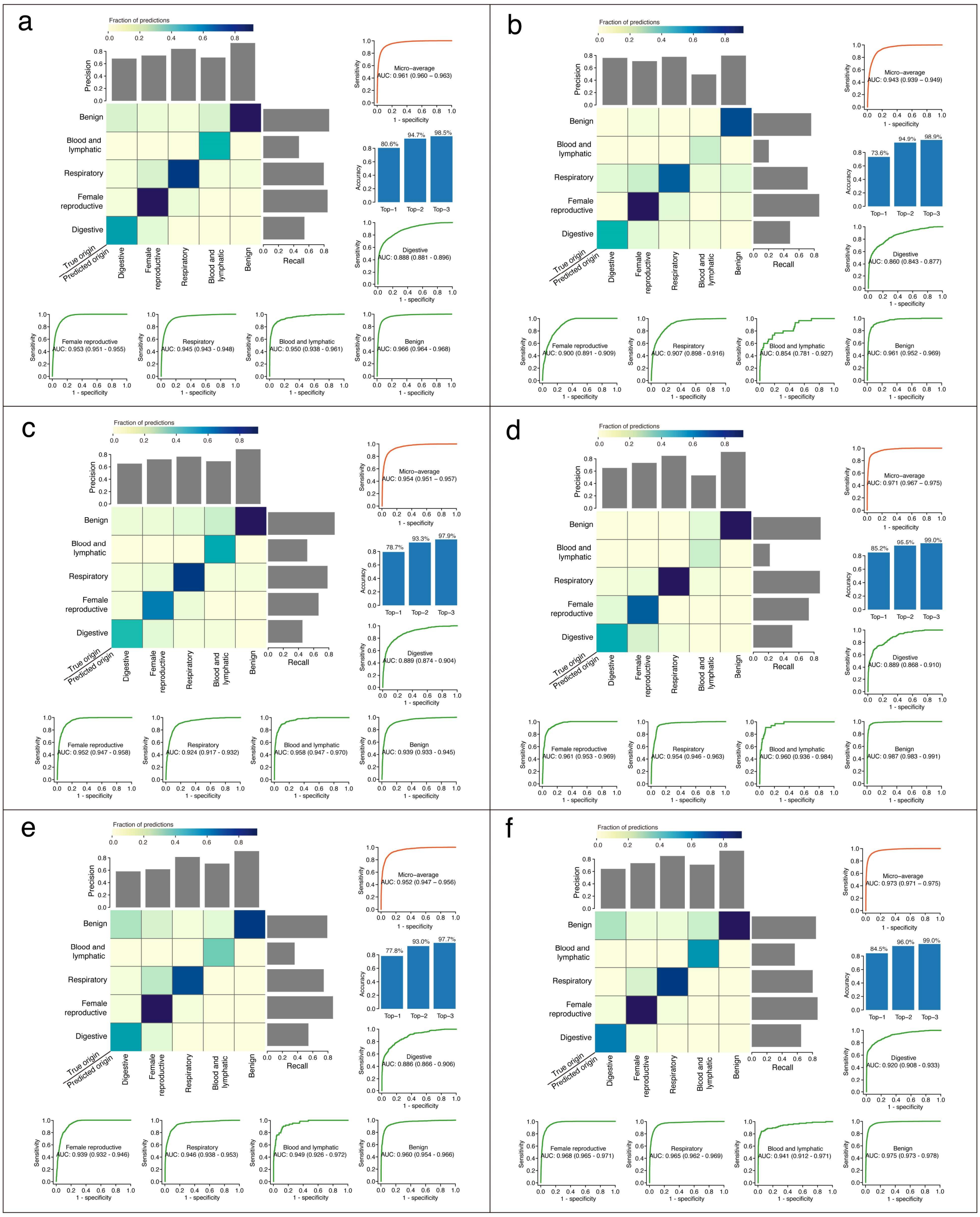

Supplementary Fig. 1a. Classification performance of attention-based multiple instance learning (AbMIL) model on testing sets by histological image feature. a, The confusion matrix, along with precision and recall is plotted for isolated tumor cells origin prediction on the combined testing sets (n = 27337). b, Tianjin testing set. c, Zhengzhou testing set. d, Suzhou testing set. e, Tianjin-P testing set. f, Yantai testing set.

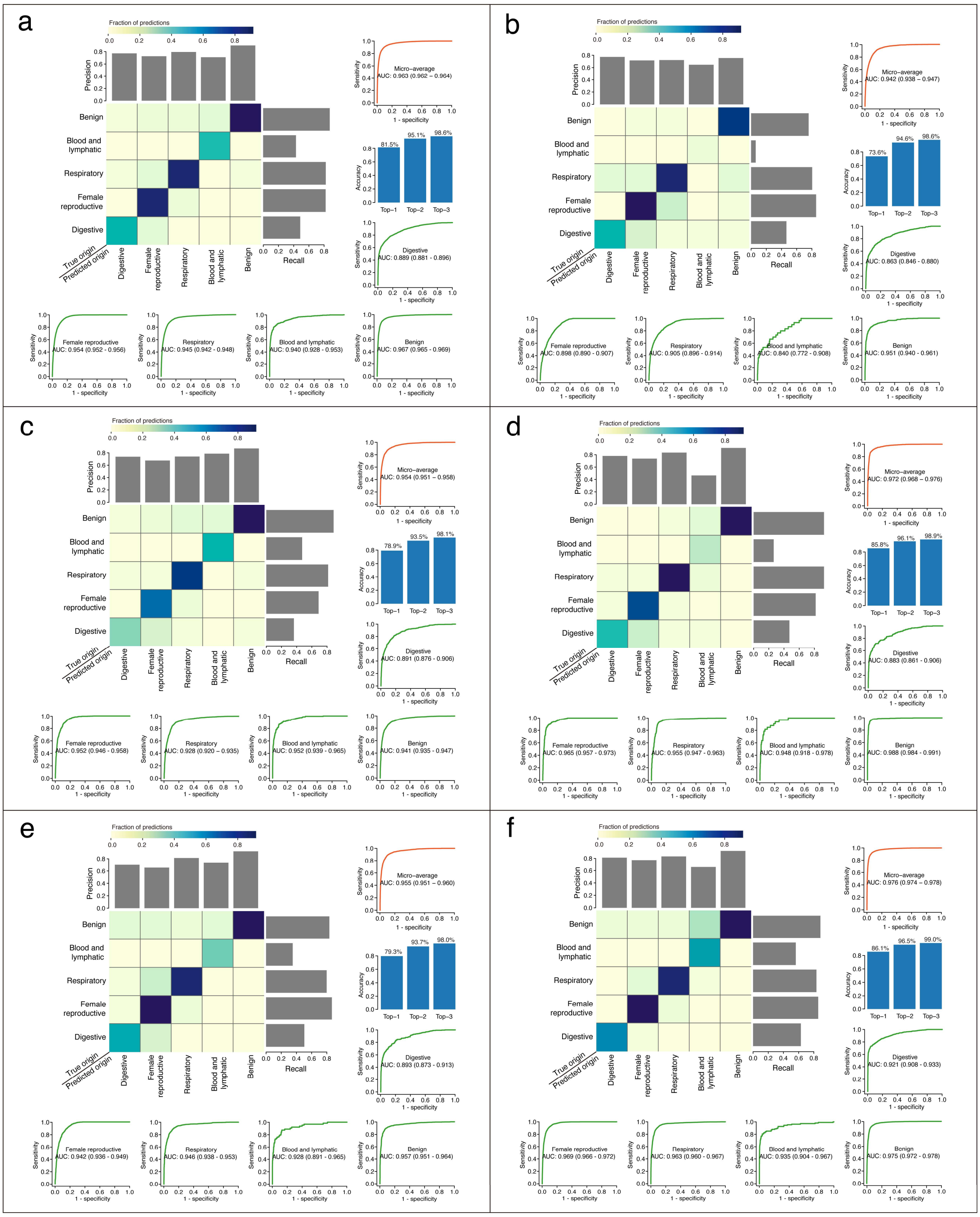

Supplementary Fig. 1b. Classification performance of attention-based multiple instance learning (AbMIL) model on testing sets by cytological image feature. a, The confusion matrix, along with precision and recall is plotted for isolated tumor cells origin prediction on the combined testing sets (n = 27337). b, Tianjin testing set. c, Zhengzhou testing set. d, Suzhou testing set. e, Tianjin-P testing set. f, Yantai testing set.

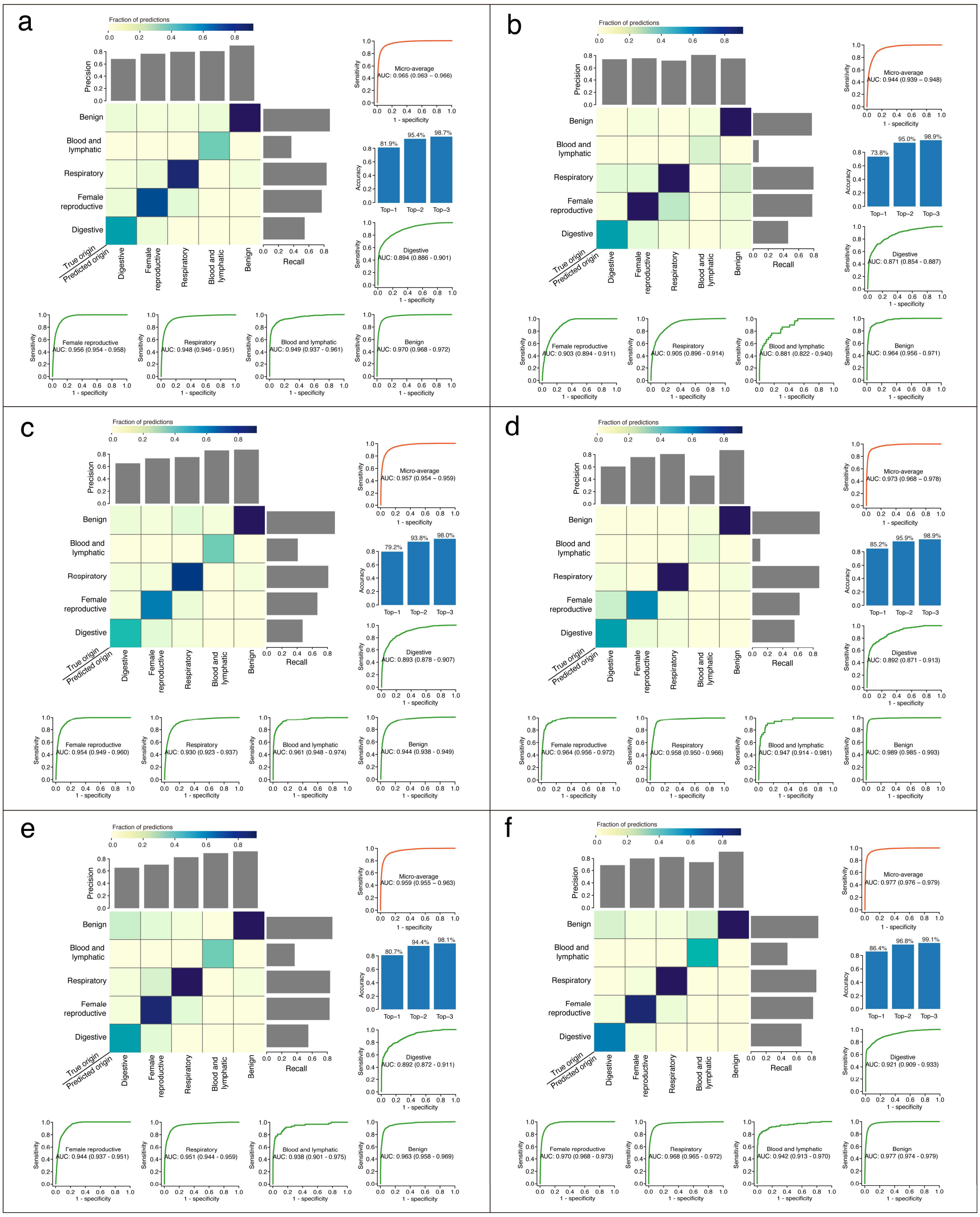

Supplementary Fig. 1c. Classification performance of attention-based multiple instance learning (AbMIL) model on testing sets by cytological and histological image feature. a, The confusion matrix, along with precision and recall is plotted for isolated tumor cells origin prediction on the combined testing sets (n = 27337). b, Tianjin testing set. c, Zhengzhou testing set. d, Suzhou testing set. e, Tianjin-P testing set. f, Yantai testing set.

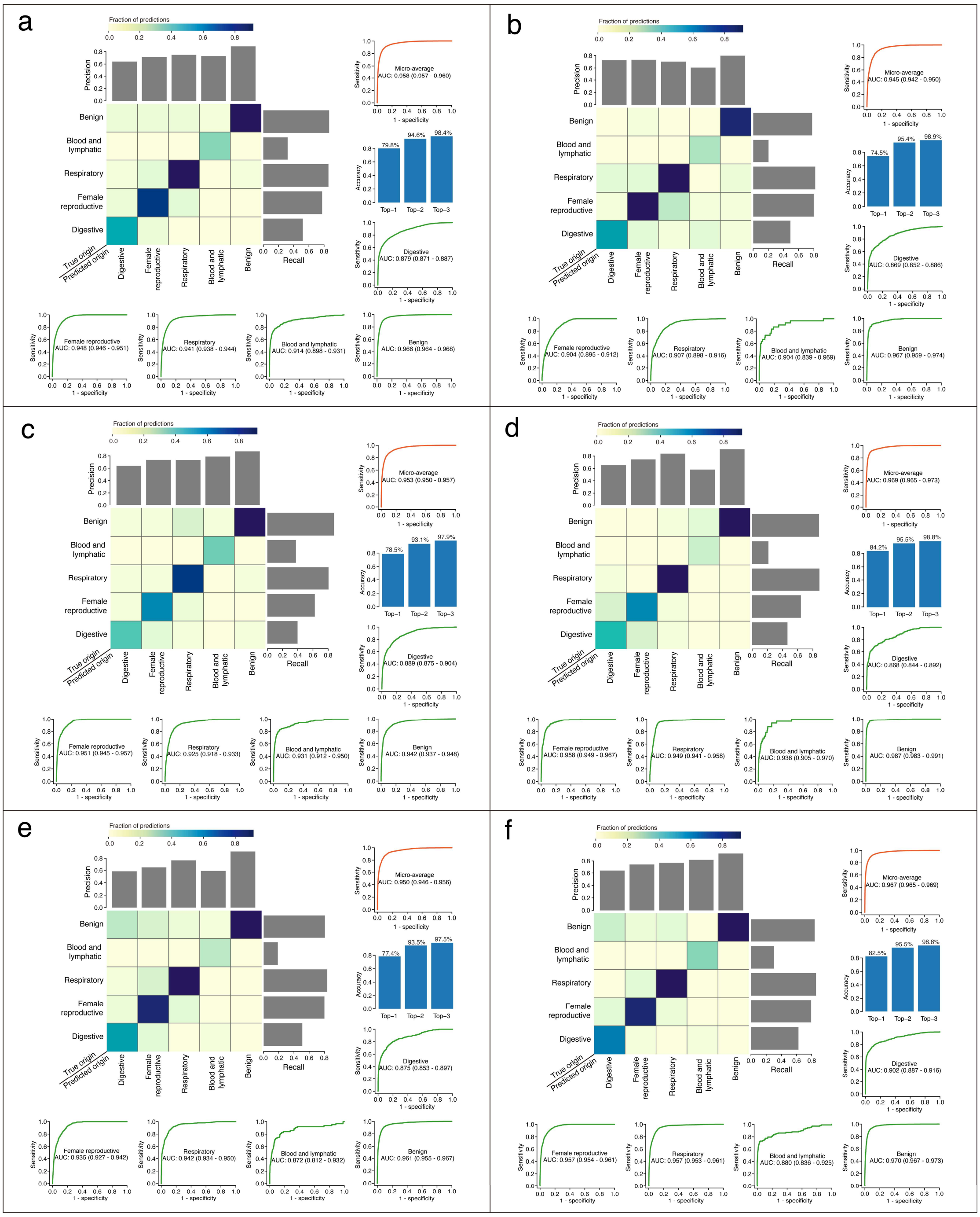

Supplementary Fig. 2a. Classification performance of AbMIL with multiple attention branches (AbMIL-MB) model on testing sets by histological image feature. a, The confusion matrix, along with precision and recall is plotted for isolated tumor cells origin prediction on the combined testing sets (n = 27337). b, Tianjin testing set. c, Zhengzhou testing set. d, Suzhou testing set. e, Tianjin-P testing set. f, Yantai testing set.

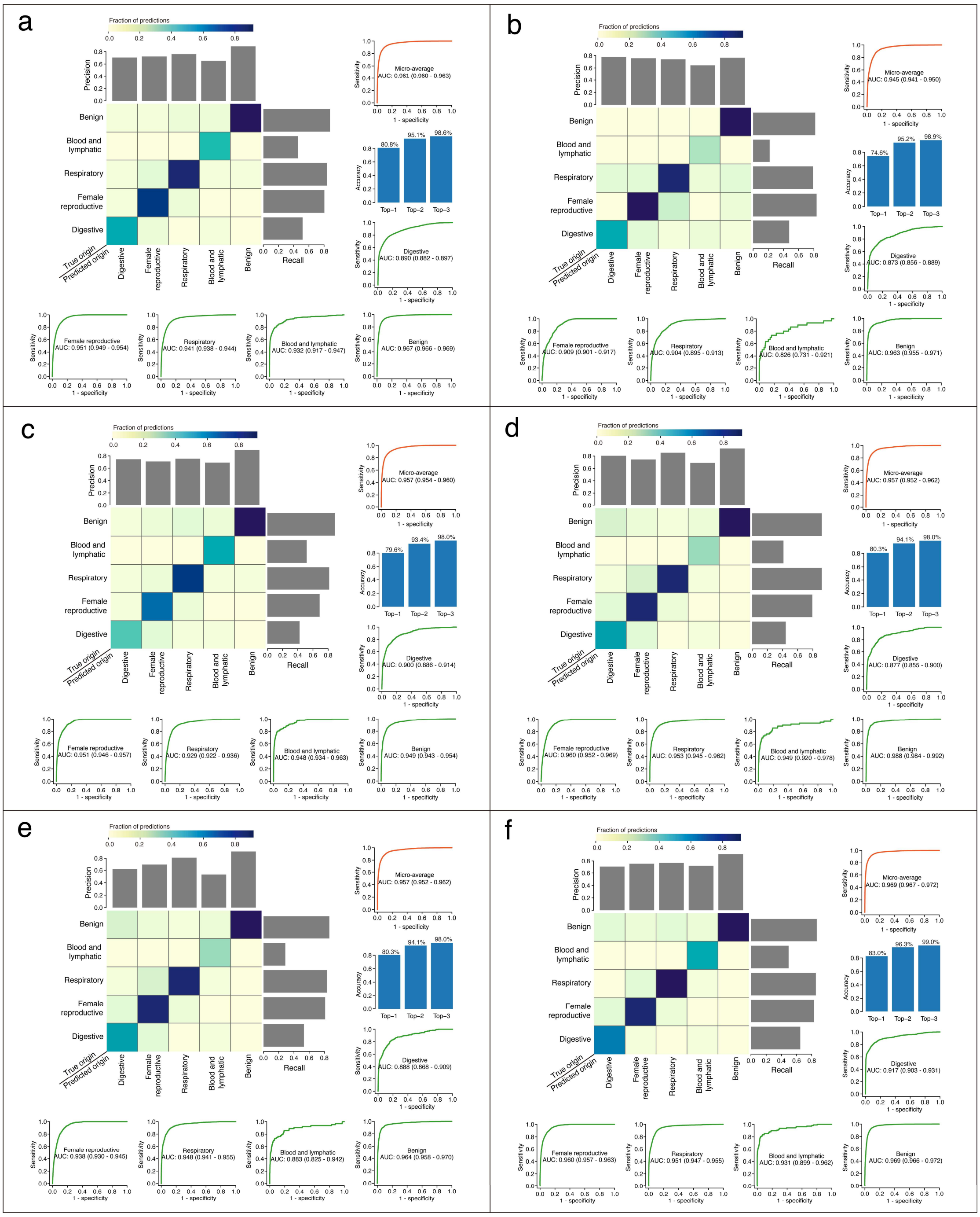

Supplementary Fig. 2b. Classification performance of AbMIL with multiple attention branches (AbMIL-MB) model on testing sets by cytological image feature. a, The confusion matrix, along with precision and recall is plotted for isolated tumor cells origin prediction on the combined testing sets (n = 27337). b, Tianjin testing set. c, Zhengzhou testing set. d, Suzhou testing set. e, Tianjin-P testing set. f, Yantai testing set.

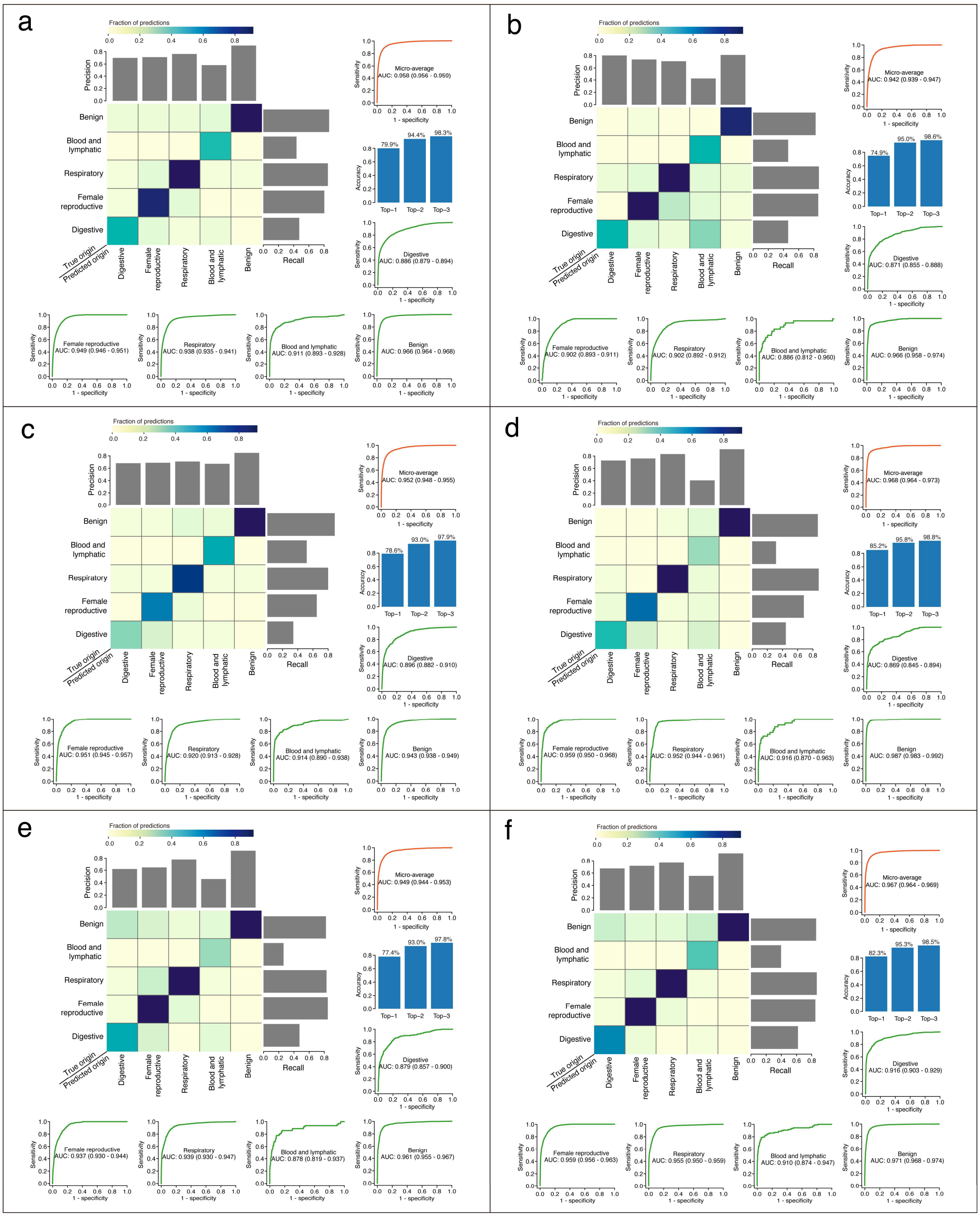

Supplementary Fig. 2c. Classification performance of AbMIL with multiple attention branches (AbMIL-MB) model on testing sets by cytological and histological image feature. a, The confusion matrix, along with precision and recall is plotted for isolated tumor cells origin prediction on the combined testing sets (n = 27337). b, Tianjin testing set. c, Zhengzhou testing set. d, Suzhou testing set. e, Tianjin-P testing set. f, Yantai testing set.

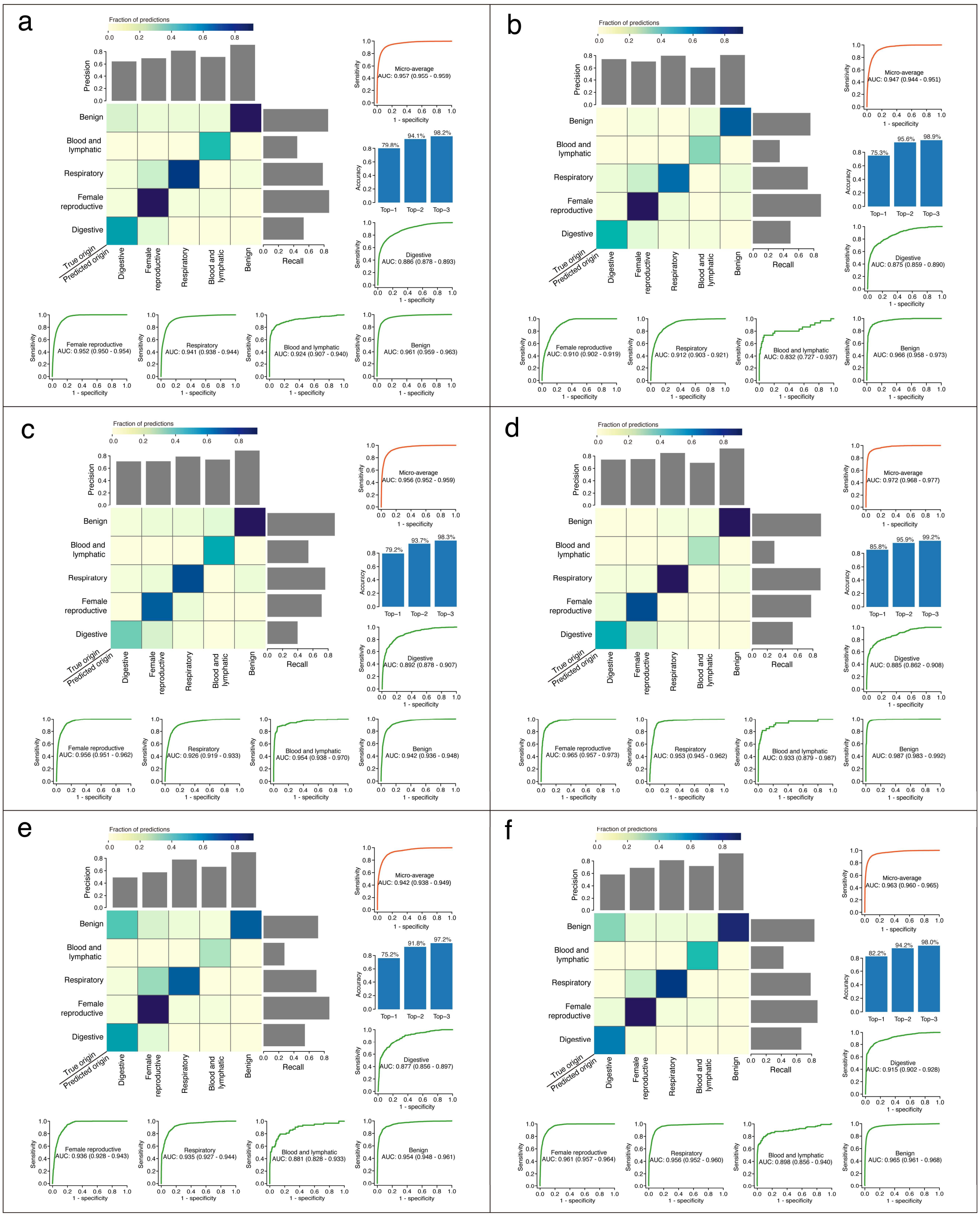

Supplementary Fig. 3a. Classification performance of Transformer-based multiple-instance learning (TransMIL) model on testing sets by histological image feature. a, The confusion matrix, along with precision and recall is plotted for isolated tumor cells origin prediction on the combined testing sets (n = 27337). b, Tianjin testing set. c, Zhengzhou testing set. d, Suzhou testing set. e, Tianjin-P testing set. f, Yantai testing set.

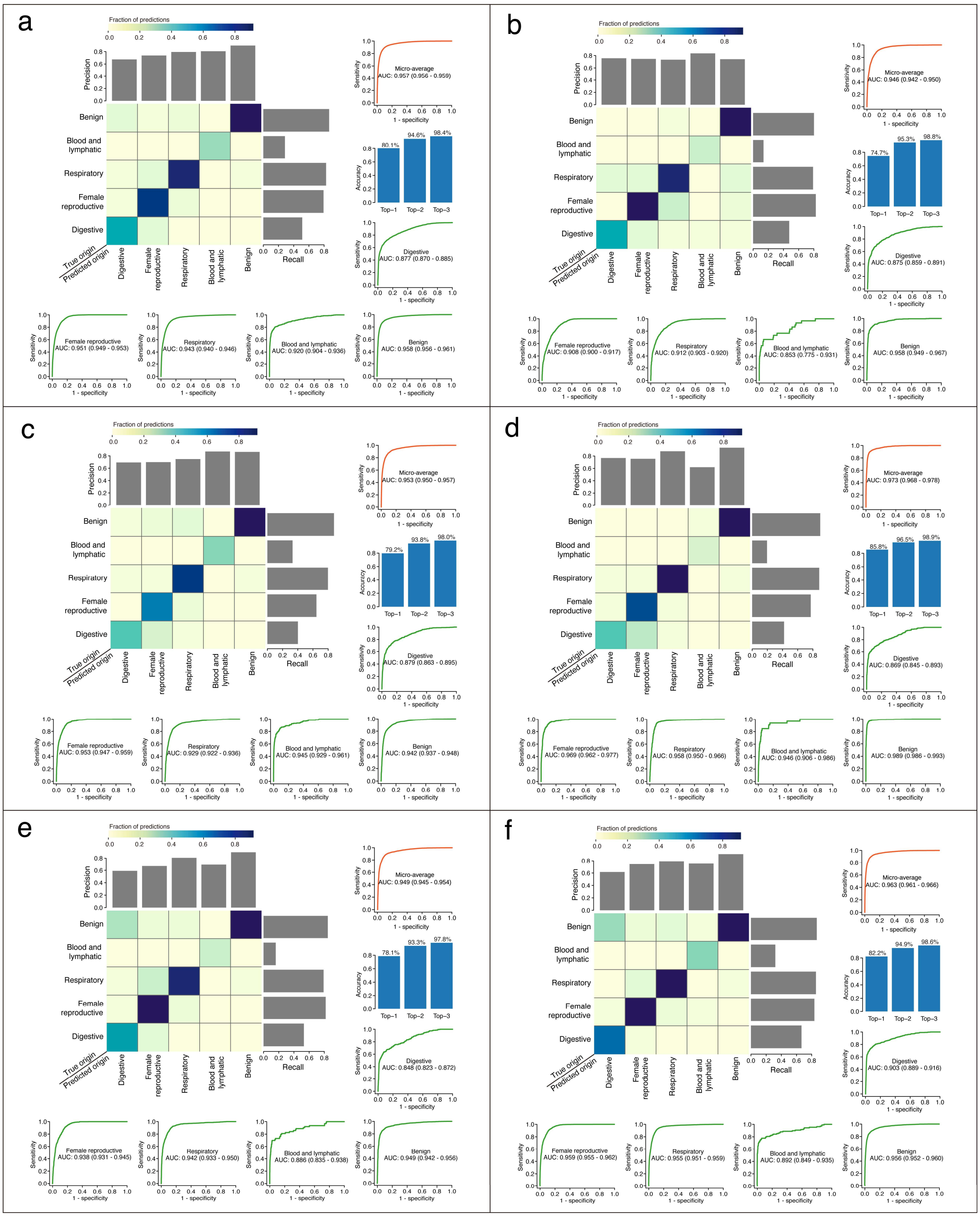

Supplementary Fig. 3b. Classification performance of Transformer-based multiple-instance learning (TransMIL) model on testing sets by cytological image feature. a, The confusion matrix, along with precision and recall is plotted for isolated tumor cells origin prediction on the combined testing sets (n = 27337). b, Tianjin testing set. c, Zhengzhou testing set. d, Suzhou testing set. e, Tianjin-P testing set. f, Yantai testing set.

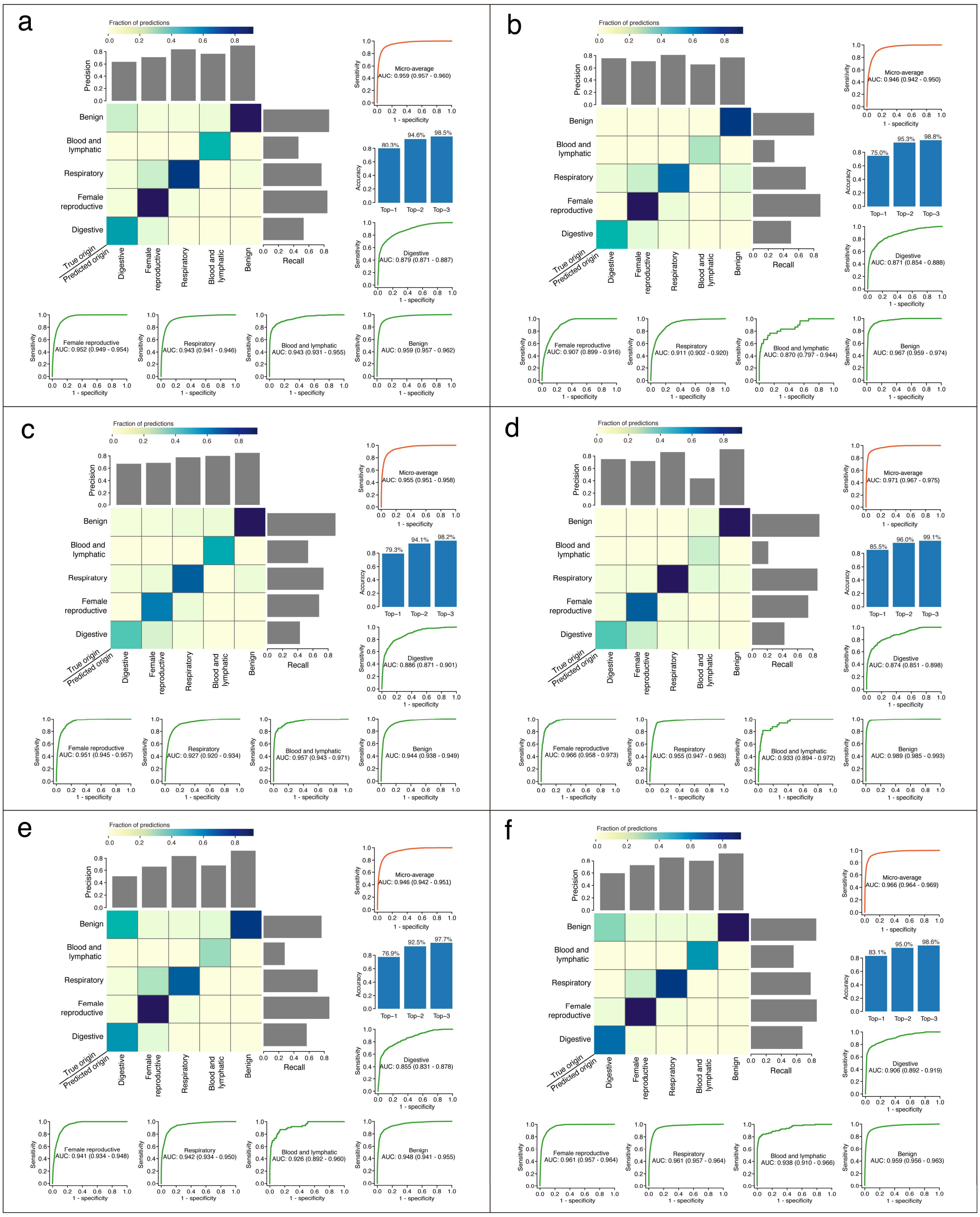

Supplementary Fig. 3c. Classification performance of Transformer-based multiple-instance learning (TransMIL) model on testing sets by cytological and histological image feature. a, The confusion matrix, along with precision and recall is plotted for isolated tumor cells origin prediction on the combined testing sets (n = 27337). b, Tianjin testing set. c, Zhengzhou testing set. d, Suzhou testing set. e, Tianjin-P testing set. f, Yantai testing set.

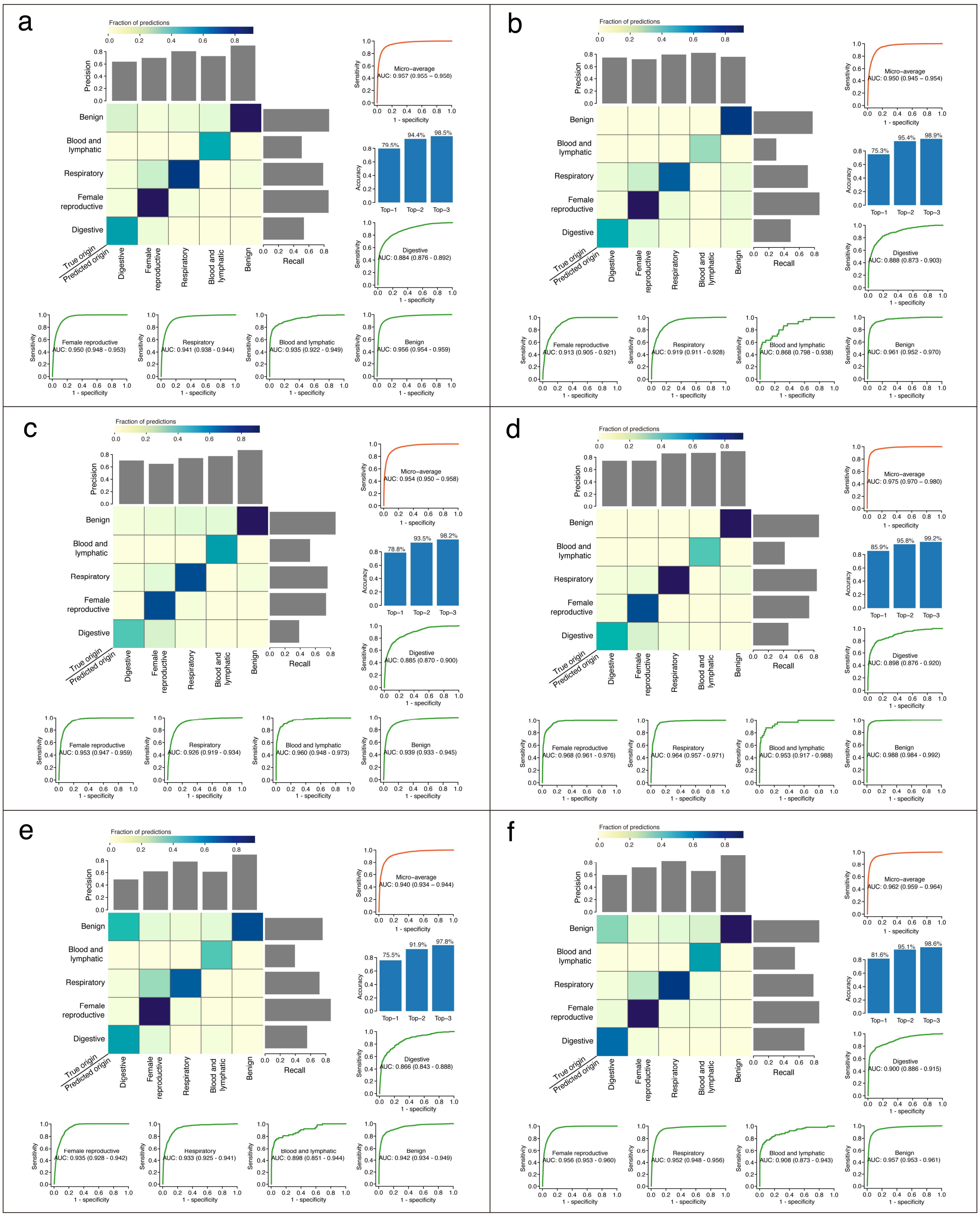

Supplementary Fig. 4a. Classification performance of TransMIL with cross-modality attention model on testing sets by histological image feature. a, The confusion matrix, along with precision and recall is plotted for isolated tumor cells origin prediction on the combined testing sets (n = 27337). b, Tianjin testing set. c, Zhengzhou testing set. d, Suzhou testing set. e, Tianjin-P testing set. f, Yantai testing set.

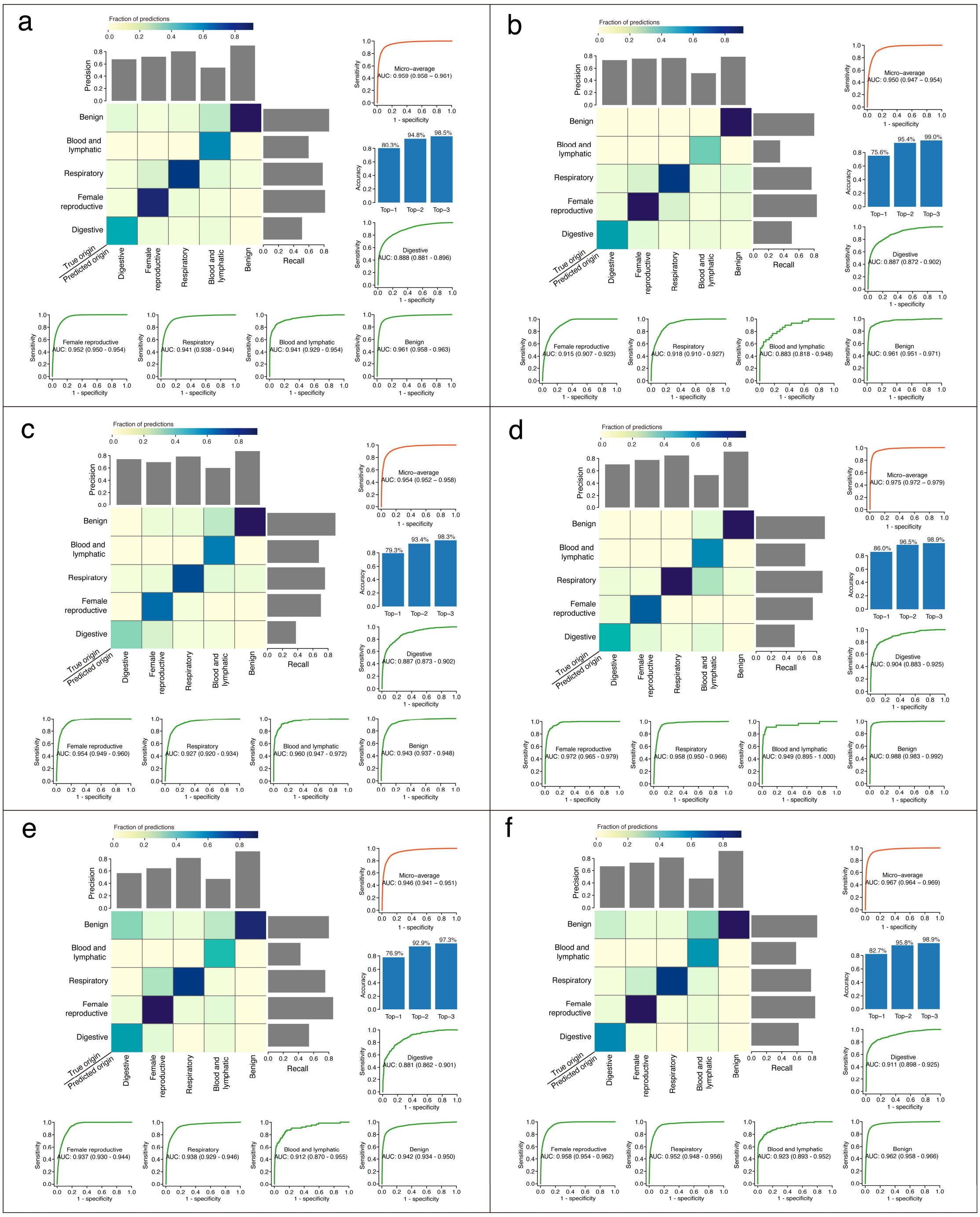

Supplementary Fig. 4b. Classification performance of TransMIL with cross-modality attention model on testing sets by cytological image feature. a, The confusion matrix, along with precision and recall is plotted for isolated tumor cells origin prediction on the combined testing sets (n = 27337). b, Tianjin testing set. c, Zhengzhou testing set. d, Suzhou testing set. e, Tianjin-P testing set. f, Yantai testing set.

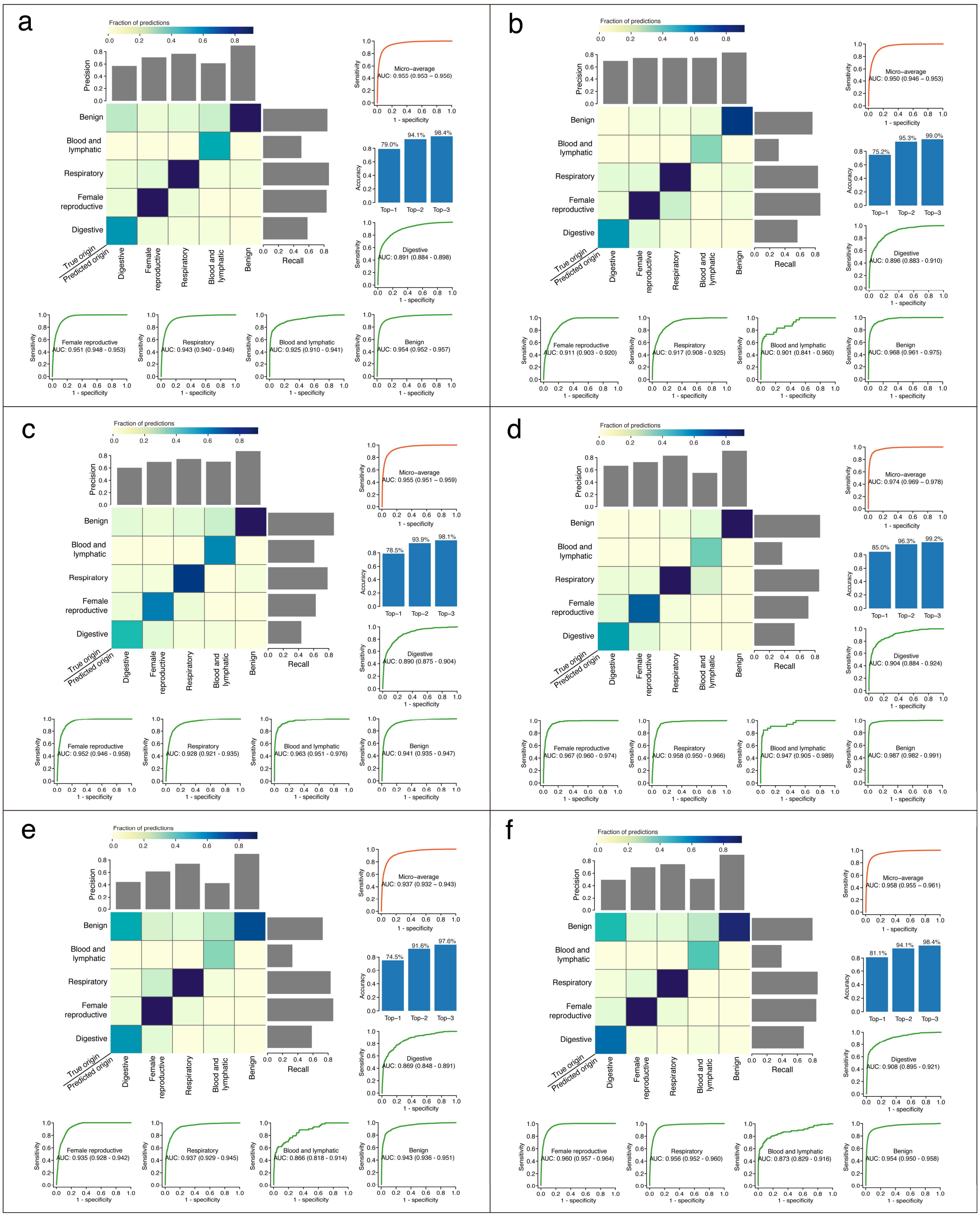

Supplementary Fig. 4c. Classification performance of TransMIL with cross-modality attention model on testing sets by cytological and histological image feature. a, The confusion matrix, along with precision and recall is plotted for isolated tumor cells origin prediction on the combined testing sets (n = 27337). b, Tianjin testing set. c, Zhengzhou testing set. d, Suzhou testing set. e, Tianjin-P testing set. f, Yantai testing set.

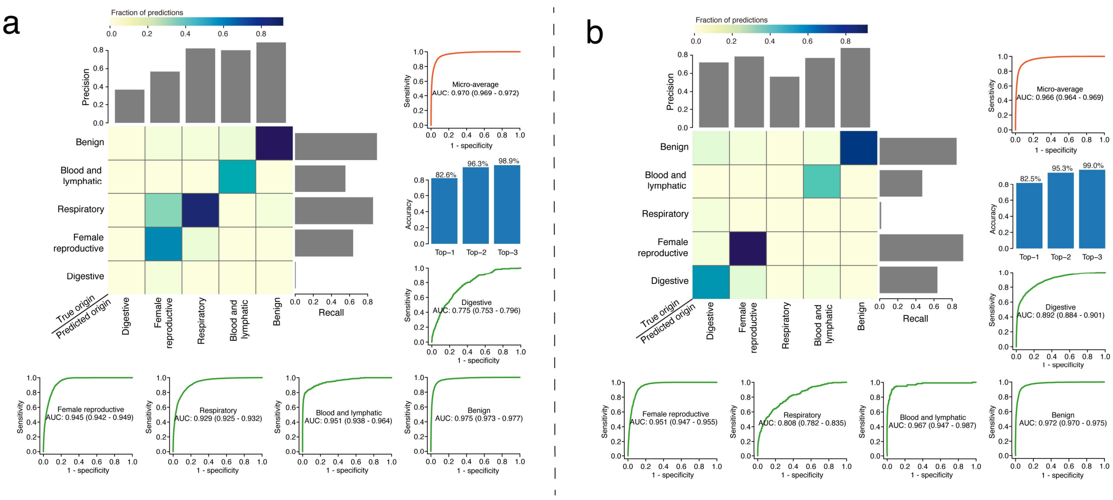

Supplementary Fig. 5. Classification performance of TORCH model on hydrothorax and ascites independently. Among five categories, TORCH achieved higher AUROC values in ascites (b) than hydrothorax (a) for digestive (0.892 versus 0.775; *P*<0.001), female reproductive (0.951 versus 0.945; *P*=0.012) systems and lower AUROC value for respiratory system (0.808 versus 0.929; *P*<0.001). No significant differences were observed for benign diseases (0.972 versus 0.975; P =0.068) and blood and lymphatic system (0.967 versus 0.951; *P*=0.122) in ascites versus hydrothorax.

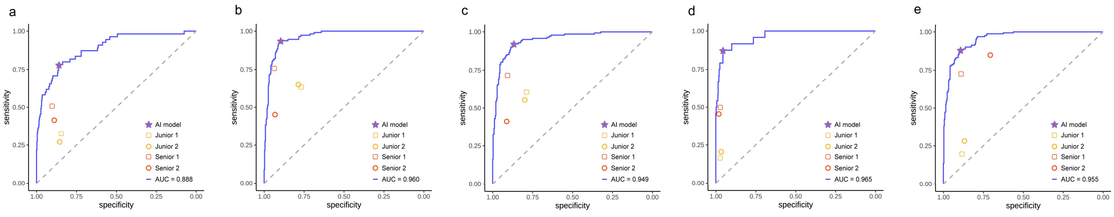

Supplementary Fig. 6. Classification performance of TORCH model and four pathologists on the five categories. a-e, digestive group, female reproductive group, respiratory group, blood and lymphatic group, benign group. Within an independent testing dataset of 495 cytological smear images, as compared with pathologists, AI model achieved an overall significantly higher accuracy (Mean: 0.896 versus 0.813; *P*=0.038), sensitivity (Mean: 0.880 versus 0.485; *P*<0.001), and precision (Mean: 0.634 versus 0.486; *P*<0.001).

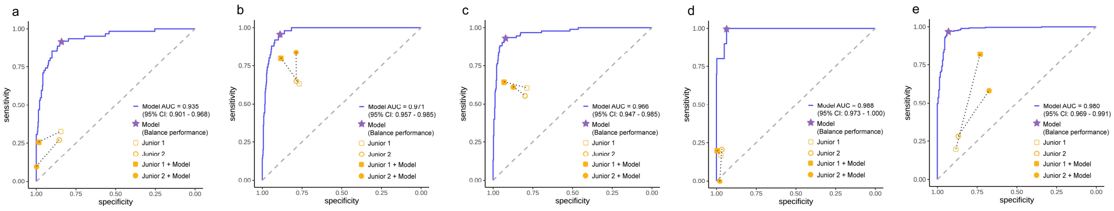

Supplementary Fig. 7. Classification performance of junior pathologists with AI assistance on the five categories. a-e, digestive group, female reproductive group, respiratory group, blood and lymphatic group, benign group. Within an independent testing dataset of 496 cytological smear images, by TORCH model assistance, junior pathologists achieved significantly higher overall top-1 accuracy as compared with junior pathologists without TORCH [62.3% (95% CI: 59.3-64.9%) versus 43.3% (40.0-46.0%); Permutation test, *P*<0.001]. Among these five categories, accuracies of these two junior pathologists on digestive system had been improved most (*P*=0.032).

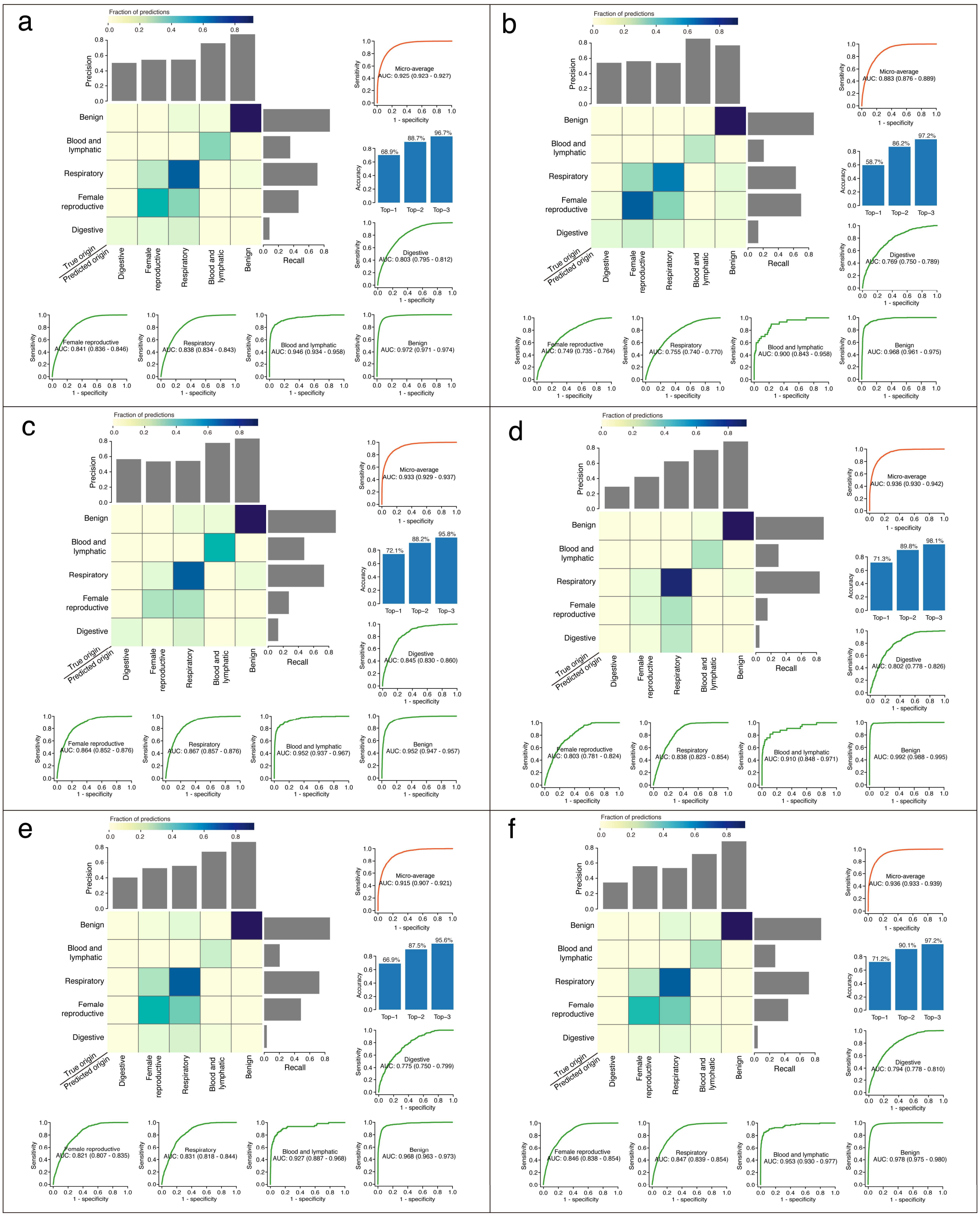

Supplementary Fig. 8. Classification performance of TORCH with ablation. For TORCH with ablation, the confusion matrix, along with precision and recall is also plotted on the overall five testing sets (n =27337). Results showed that ablating sex, age and specimen sampling site led to a substantial decrease in AUROC (0.969 versus 0.925; *P*<0.001) and top-1 accuracy (82.6% versus 68.9%; *P*<0.001), top-2 accuracy (95.9% versus 88.7%; *P*<0.001). Among these five categories on the combined 27337 dataset, AUROC values were also significantly decreased for digestive (0.904 versus 0.803; *P* <0.001], female reproductive (0.960 versus 0.841; *P*<0.001), respiratory (0.953 versus 0.838; *P* <0.001), blood and lymphatic (0.957 versus 0.946; *P* < 0.001) systems and benign diseases (0.974 versus 0.972; *P*=0.020). a, combined 27337 dataset. b, Tianjin testing set. c, Zhengzhou testing set. d, Suzhou testing set. e, Tianjin-P testing set. f, Yantai testing set.

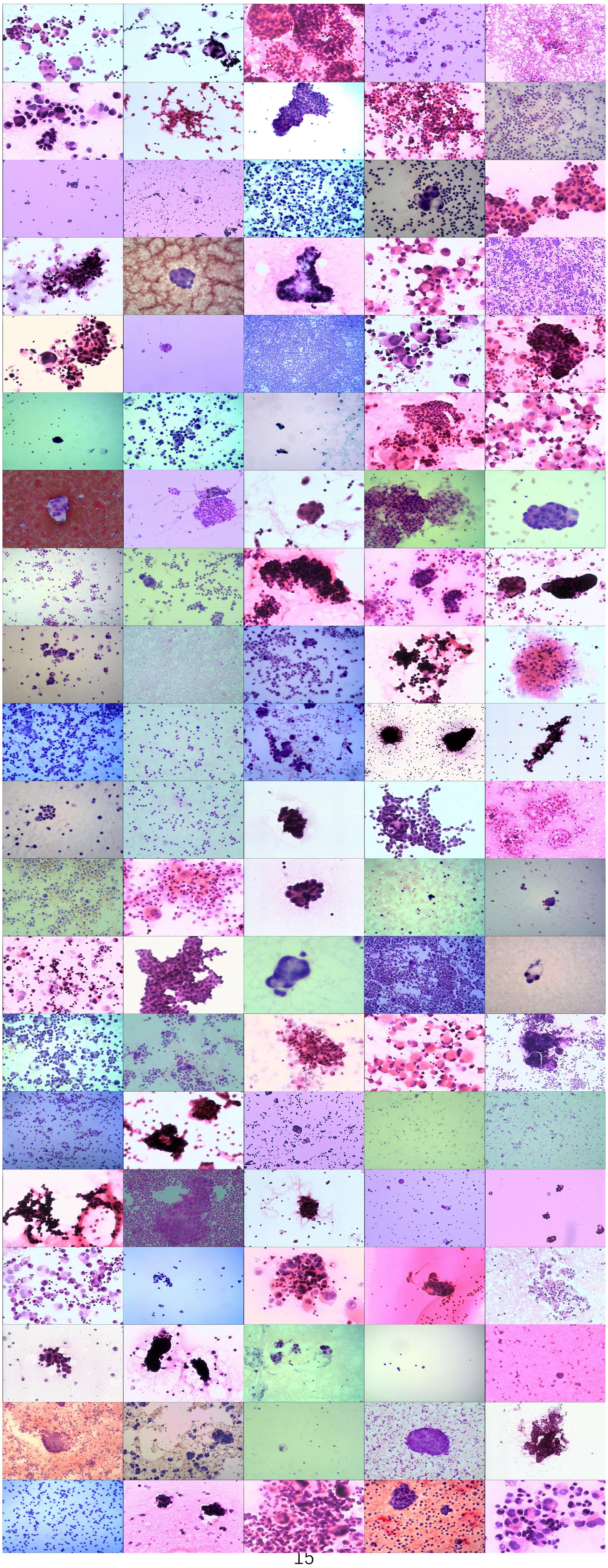

Supplementary Fig. 9. Falsely classified malignant cases. A considerable number of falsely classified images are due to insufficient number of tumor cells captured or more impurities in the cytological image.

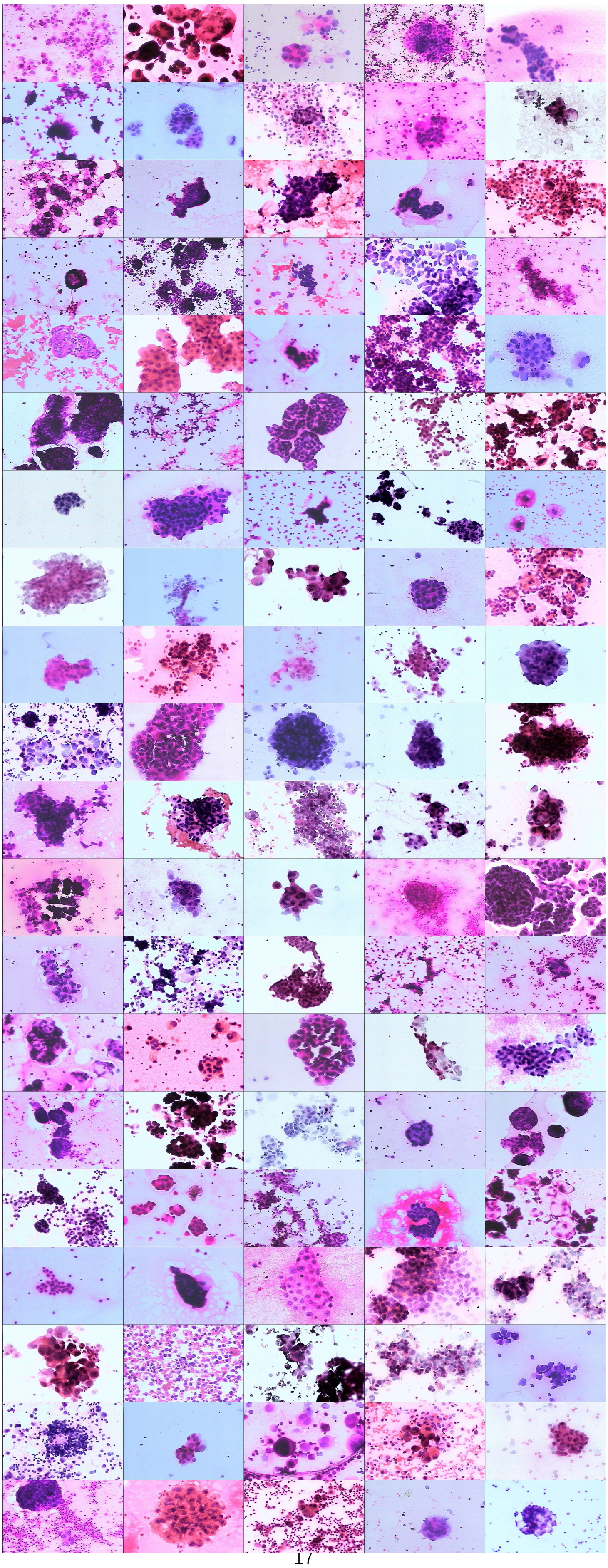

Supplementary Fig. 10. Exemplified correctly classified malignant cases. Most images are brightly colored, with sufficient cells and clean background.

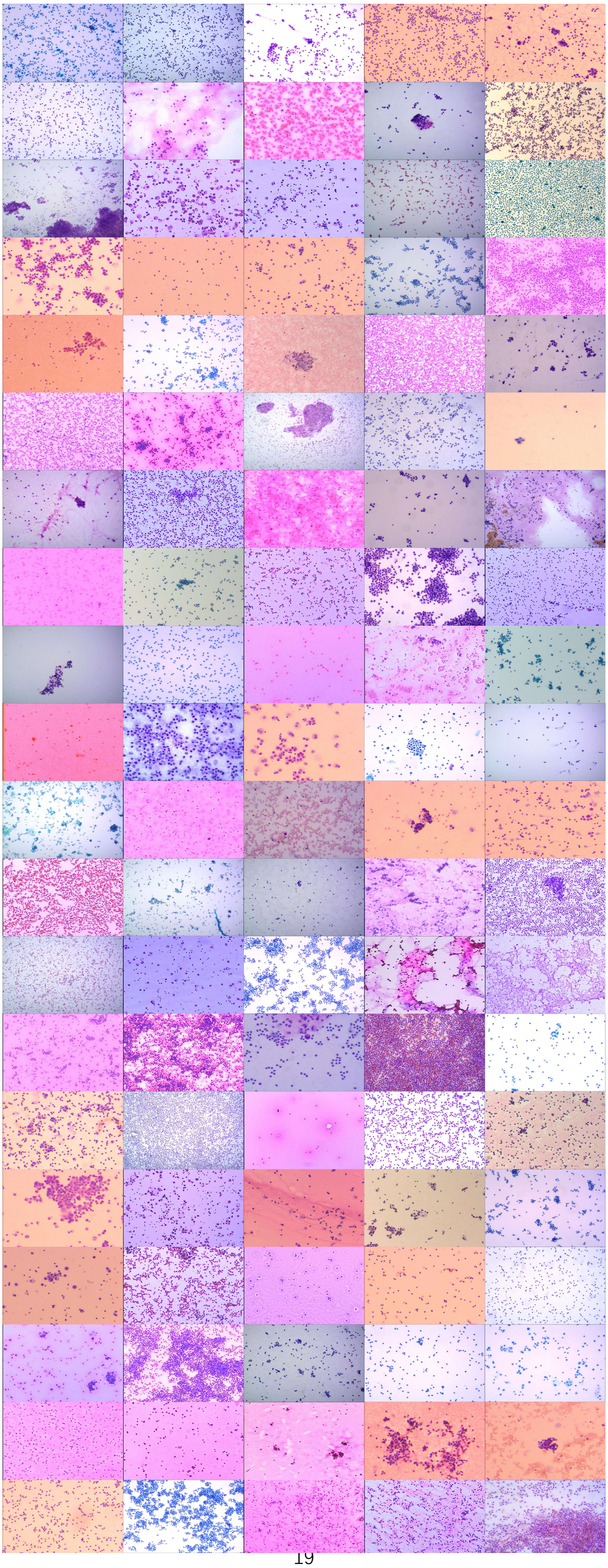

Supplementary Fig. 11. Exemplified correctly classified benign cases. Compared with malignant cases, benign images are mainly composed of inflammatory cells such as macrophages, lymphocytes and mesothelial cells. These cells are often scattered and do not have large nucleoli.

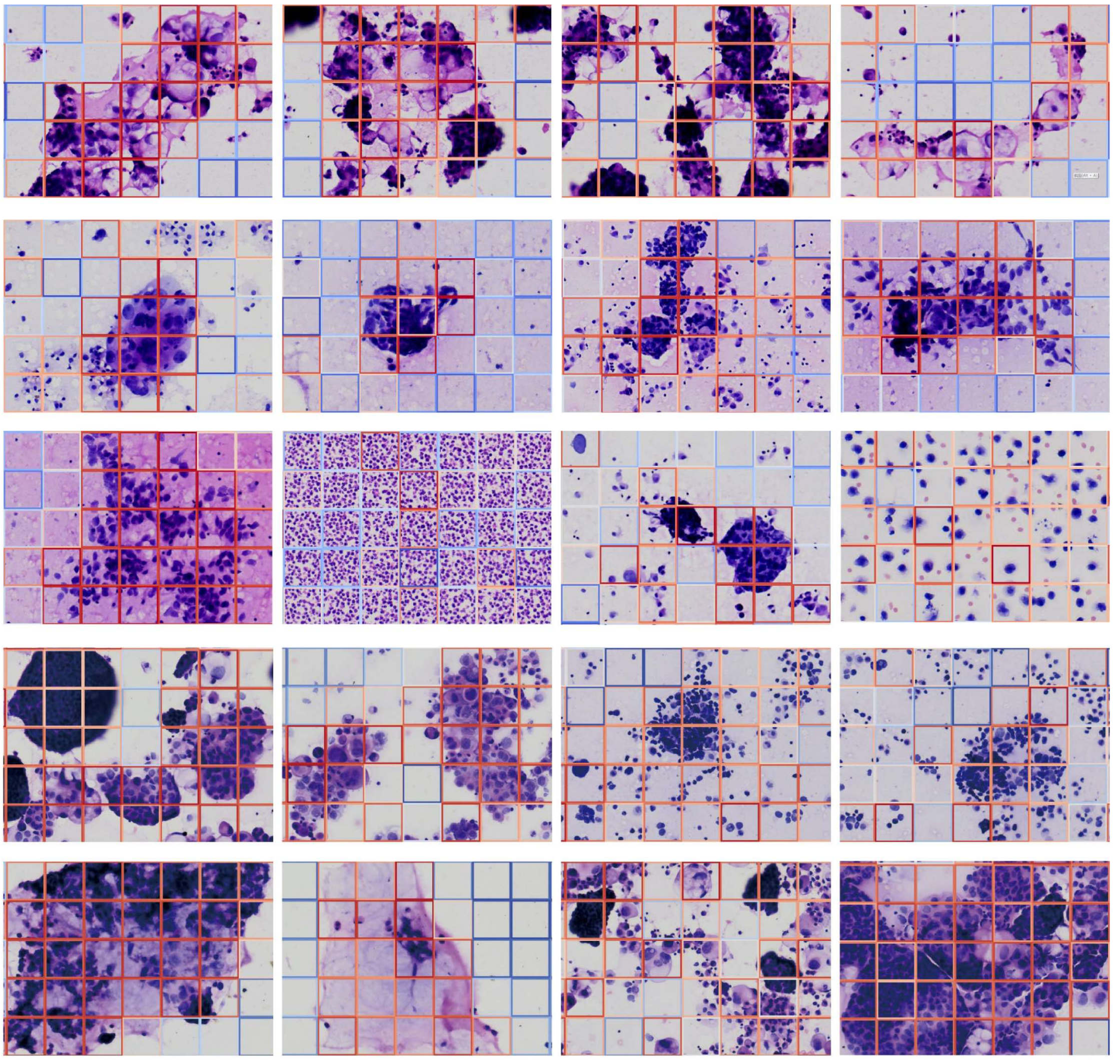

Supplementary Fig. 12. Examples of haematoxylin-eosin staining cytological attention heatmaps. The frame of each square is marked with different colors. Red frame indicates that a region is highly informative for the classification decision making and blue frame indicates that the region has lower diagnostic value.

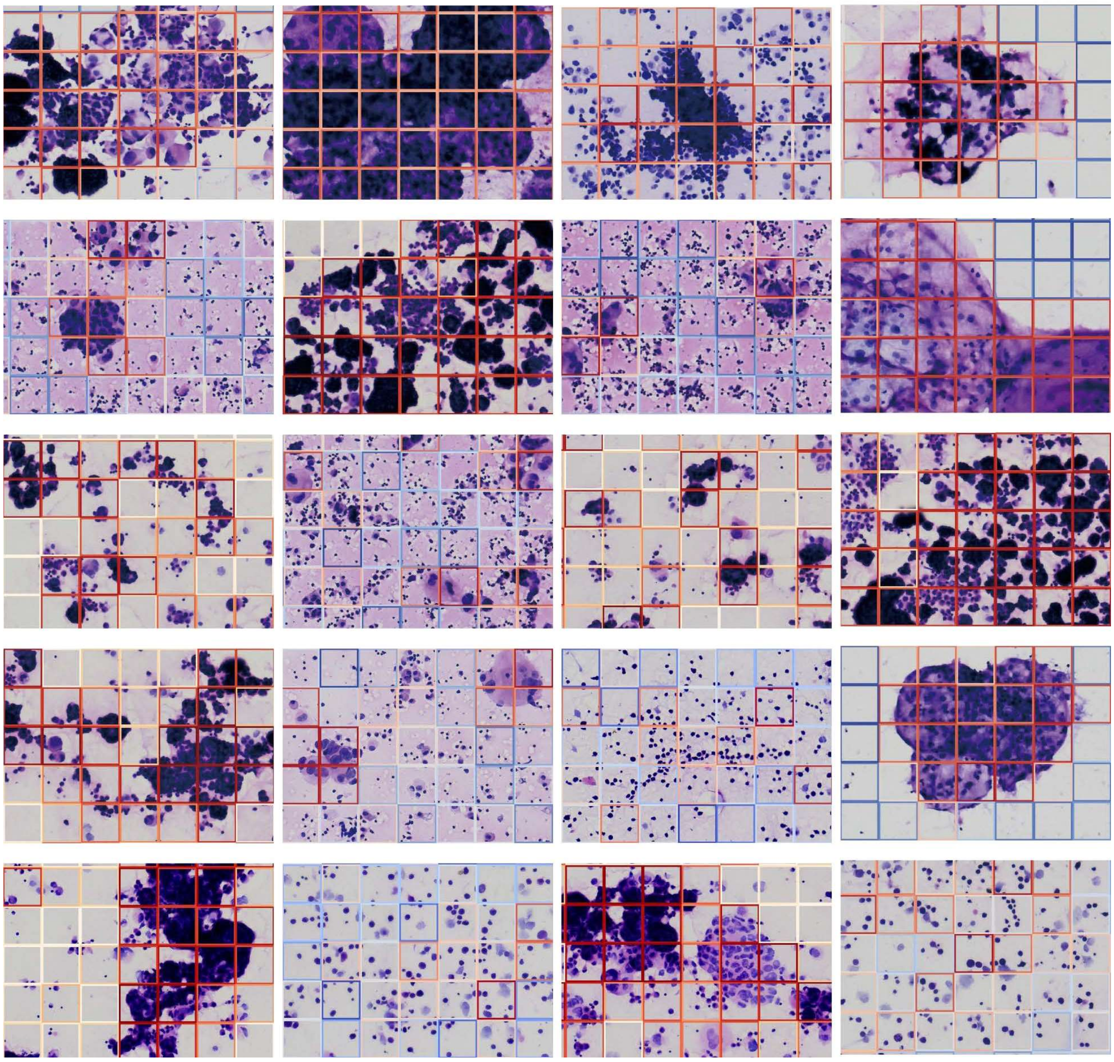

Supplementary Fig. 13. Examples of haematoxylin-eosin staining cytological attention heatmaps. The frame of each square is marked with different colors. Red frame indicates that a region is highly informative for the classification decision making and blue frame indicates that the region has lower diagnostic value.

Supplementary Table 5a. Classification performance of TORCH model on Tianjin testing set.

Performance Metrics	Digestive Syst.	Female reproductive Syst.	Respiratory Syst.	Blood and lymphatic Syst.	Benign
Accuracy (95% CI)	0.836 (0.825 - 0.847)	0.827 (0.815 - 0.838)	0.828 (0.816 - 0.839)	0.913 (0.904 - 0.921)	0.922 (0.914 - 0.930)
Sensitivity (95% CI)	0.782 (0.746 - 0.814)	0.886 (0.870 - 0.901)	0.877 (0.859 - 0.894)	0.733 (0.541 - 0.877)	0.906 (0.876 - 0.931)
Specificity (95% CI)	0.845 (0.833 - 0.857)	0.787 (0.771 - 0.803)	0.802 (0.786 - 0.816)	0.914 (0.905 - 0.922)	0.924 (0.915 - 0.932)
Precision (95% CI)	0.454 (0.423 - 0.485)	0.733 (0.713 - 0.752)	0.697 (0.675 - 0.718)	0.058 (0.037 - 0.087)	0.602 (0.565 - 0.638)
Negative Predictive Value (95% CI)	0.959 (0.952 - 0.966)	0.913 (0.901 - 0.925)	0.926 (0.915 - 0.936)	0.998 (0.996 - 0.999)	0.987 (0.983 - 0.991)
AUROC (95% CI)	0.896 (0.882 - 0.909)	0.917 (0.909 - 0.925)	0.921 (0.913 - 0.929)	0.891 (0.829 - 0.953)	0.971 (0.965 - 0.978)
Case number	591	1662	1433	30	470

Supplementary Table 5b. Classification performance of TORCH model on Zhengzhou testing set.

Performance Metrics	Digestive Syst.	Female reproductive Syst.	Respiratory Syst.	Blood and lymphatic Syst.	Benign
Accuracy (95% CI)	0.866 (0.858 - 0.875)	0.891 (0.883 - 0.898)	0.873 (0.865 - 0.881)	0.902 (0.895 - 0.910)	0.893 (0.885 - 0.900)
Sensitivity (95% CI)	0.778 (0.740 - 0.812)	0.904 (0.881 - 0.924)	0.887 (0.870 - 0.902)	0.930 (0.887 - 0.960)	0.892 (0.881 - 0.903)
Specificity (95% CI)	0.875 (0.866 - 0.883)	0.889 (0.880 - 0.897)	0.869 (0.859 - 0.878)	0.901 (0.894 - 0.909)	0.893 (0.882 - 0.904)
Precision (95% CI)	0.372 (0.344 - 0.401)	0.536 (0.508 - 0.563)	0.698 (0.677 - 0.718)	0.251 (0.221 - 0.283)	0.893 (0.882 - 0.904)
Negative Predictive Value (95% CI)	0.976 (0.972 - 0.980)	0.985 (0.981 - 0.988)	0.957 (0.951 - 0.963)	0.997 (0.995 - 0.998)	0.892 (0.881 - 0.903)
AUROC (95% CI)	0.905 (0.891 - 0.918)	0.960 (0.955 - 0.965)	0.938 (0.932 - 0.944)	0.969 (0.958 - 0.980)	0.953 (0.948 - 0.958)
Case number	544	774	1589	214	3113

Supplementary Table 5c. Classification performance of TORCH model on Suzhou testing set.

Performance Metrics	Digestive Syst.	Female reproductive Syst.	Respiratory Syst.	Blood and lymphatic Syst.	Benign
Accuracy (95% CI)	0.882 (0.869 - 0.895)	0.912 (0.900 - 0.923)	0.922 (0.911 - 0.933)	0.951 (0.941 - 0.959)	0.965 (0.957 - 0.972)
Sensitivity (95% CI)	0.778 (0.723 - 0.826)	0.934 (0.901 - 0.958)	0.947 (0.931 - 0.960)	0.909 (0.757 - 0.981)	0.973 (0.959 - 0.983)
Specificity (95% CI)	0.896 (0.882 - 0.908)	0.909 (0.895 - 0.921)	0.906 (0.889 - 0.920)	0.951 (0.942 - 0.960)	0.961 (0.950 - 0.970)
Precision (95% CI)	0.488 (0.440 - 0.537)	0.623 (0.579 - 0.666)	0.871 (0.849 - 0.891)	0.208 (0.145 - 0.284)	0.925 (0.905 - 0.942)
Negative Predictive Value (95% CI)	0.969 (0.961 - 0.976)	0.988 (0.982 - 0.993)	0.962 (0.950 - 0.971)	0.999 (0.996 - 1.000)	0.986 (0.979 - 0.992)
AUROC (95% CI)	0.912 (0.892 - 0.931)	0.974 (0.968 - 0.981)	0.965 (0.958 - 0.972)	0.973 (0.948 - 0.998)	0.991 (0.988 - 0.994)
Case number	270	331	958	33	787

Supplementary Table 5d. Classification performance of TORCH model on Tianjin-P testing set.

Performance Metrics	Digestive Syst.	Female reproductive Syst.	Respiratory Syst.	Blood and lymphatic Syst.	Benign
Accuracy (95% CI)	0.786 (0.773 - 0.799)	0.864 (0.853 - 0.874)	0.886 (0.876 - 0.896)	0.852 (0.840 - 0.863)	0.915 (0.905 - 0.923)
Sensitivity (95% CI)	0.835 (0.789 - 0.874)	0.920 (0.900 - 0.937)	0.899 (0.880 - 0.916)	0.889 (0.784 - 0.954)	0.929 (0.915 - 0.941)
Specificity (95% CI)	0.782 (0.768 - 0.795)	0.847 (0.834 - 0.860)	0.881 (0.868 - 0.893)	0.851 (0.840 - 0.862)	0.905 (0.893 - 0.917)
Precision (95% CI)	0.250 (0.224 - 0.277)	0.637 (0.610 - 0.664)	0.754 (0.730 - 0.777)	0.089 (0.068 - 0.114)	0.862 (0.845 - 0.879)
Negative Predictive Value (95% CI)	0.982 (0.976 - 0.986)	0.973 (0.966 - 0.979)	0.955 (0.947 - 0.963)	0.998 (0.996 - 0.999)	0.952 (0.943 - 0.961)
AUROC (95% CI)	0.892 (0.872 - 0.911)	0.947 (0.940 - 0.953)	0.949 (0.941 - 0.956)	0.937 (0.904 - 0.970)	0.966 (0.961 - 0.972)
Case number	315	888	1135	63	1532

Supplementary Table 5e. Classification performance of TORCH model on Yantai testing set.

Performance Metrics	Digestive Syst.	Female reproductive Syst.	Respiratory Syst.	Blood and lymphatic Syst.	Benign
Accuracy (95% CI)	0.901 (0.896 - 0.907)	0.907 (0.901 - 0.912)	0.909 (0.903 - 0.914)	0.923 (0.918 - 0.928)	0.938 (0.933 - 0.943)
Sensitivity (95% CI)	0.785 (0.751 - 0.815)	0.921 (0.909 - 0.932)	0.943 (0.934 - 0.951)	0.852 (0.771 - 0.913)	0.954 (0.948 - 0.960)
Specificity (95% CI)	0.909 (0.903 - 0.915)	0.903 (0.896 - 0.909)	0.896 (0.889 - 0.903)	0.924 (0.919 - 0.929)	0.926 (0.919 - 0.932)
Precision (95% CI)	0.364 (0.339 - 0.389)	0.722 (0.706 - 0.738)	0.768 (0.753 - 0.781)	0.103 (0.084 - 0.125)	0.912 (0.904 - 0.920)
Negative Predictive Value (95% CI)	0.985 (0.982 - 0.987)	0.977 (0.973 - 0.980)	0.977 (0.974 - 0.981)	0.998 (0.997 - 0.999)	0.961 (0.956 - 0.966)
AUROC (95% CI)	0.925 (0.914 - 0.937)	0.971 (0.968 - 0.974)	0.969 (0.965 - 0.972)	0.948 (0.921 - 0.974)	0.980 (0.978 - 0.983)
Case number	659	2283	2822	108	4733

Supplementary Table 19. Brief summary of four deep-learning-based MIL methods.

Methods	Characteristics
Attention-based multiple instance learning (AbMIL)	**Objective**:<br>Handles each cytological image as a bag with patches as instances. Clinical factors such as age, sex and tissue sampling site are taken as instances too. Aggregates global feature by attention mechanism.<br>**Key aspective**:<br>Learns attention scores to quantify the contribution of each instance to the bag label. Instances are assumed to be independent.
Attention-based MIL with multiple branches (AbMIL-MB)	**Objective**:<br>Extension of AbMIL with multiple attention branches for class-specific predictions. Clinical factors such as age, sex and tissue sampling site are taken as instances too.<br>**Key aspective**:<br>Learns class-specific attention scores to quantify the contribution of each instance to the bag label. Instances are assumed to be independent.
Transformer-based multiple-instance learning (TransMIL)	**Objective**:<br>Handles each cytological image as a bag with patches as instances. Clinical factors such as age, sex and tissue sampling site are taken as instances too. Learns global feature by transformer.<br>**Key aspective**:<br>Instances are not assumed to be independent and connections among them are learned via self-attention mechanism.
TransMIL with cross-modality attention	**Objective**:<br>An extension of TransMIL for multimodal data fusion with attention to interconnections. Interconnections among image patches and clinical factors such as age, sex and tissue sampling site are learned via cross-attention mechanism. Learns global feature by transformer.<br>**Key aspective**:<br>Instances are not assumed to be independent and connections among them are learned via self-attention mechanism. Uses cross-attention mechanism to learn interconnections among multimodal data types.
