# Supplementary material for: Prediction of tumor origin in cancers of unknown primary origin with cytology-based deep learning
Source: Nat Med. 2024 Apr 16;30(5):1309–19. doi: 10.1038/s41591-024-02915-w (PMC11108774; doi:10.1038/s41591-024-02915-w)
Supplement: Supplementary file 2 — Reporting Summary [file 41591_2024_2915_MOESM2_ESM.pdf]

nature portfolio

Corresponding author(s): Xiangchun Li

Last updated by author(s): Feb 22, 2024

Reporting Summary

Nature Portfolio wishes to improve the reproducibility of the work that we publish. This form provides structure for consistency and transparency in reporting. For further information on Nature Portfolio policies, see our Editorial Policies and the Editorial Policy Checklist.

Statistics

For all statistical analyses, confirm that the following items are present in the figure legend, table legend, main text, or Methods section.

n/a	Confirmed	
☐	☒	The exact sample size (*n*) for each experimental group/condition, given as a discrete number and unit of measurement
☐	☒	A statement on whether measurements were taken from distinct samples or whether the same sample was measured repeatedly
☐	☒	The statistical test(s) used AND whether they are one- or two-sided<br>*Only common tests should be described solely by name; describe more complex techniques in the Methods section.*
☐	☒	A description of all covariates tested
☐	☒	A description of any assumptions or corrections, such as tests of normality and adjustment for multiple comparisons
☐	☒	A full description of the statistical parameters including central tendency (e.g. means) or other basic estimates (e.g. regression coefficient) AND variation (e.g. standard deviation) or associated estimates of uncertainty (e.g. confidence intervals)
☐	☒	For null hypothesis testing, the test statistic (e.g. *F*, *t*, *r*) with confidence intervals, effect sizes, degrees of freedom and *P* value noted<br>*Give P values as exact values whenever suitable.*
☐	☒	For Bayesian analysis, information on the choice of priors and Markov chain Monte Carlo settings
☐	☒	For hierarchical and complex designs, identification of the appropriate level for tests and full reporting of outcomes
☐	☒	Estimates of effect sizes (e.g. Cohen's *d*, Pearson's *r*), indicating how they were calculated

Our web collection on statistics for biologists contains articles on many of the points above.

Software and code

Policy information about availability of computer code

Data collection	Whole slide images were collected from TCGA (The Cancer Genome Atlas) via the NIH Genomic Data Commons Data Portal. Cytological data were collected from cohorts of patients who attended four large tertiary referral institutions. Cytological smear images were acquired by microscope (JVC TK-C9501EC, Olympus BX51) at 400× or 200× equivalent magnification.We used the self-supervised feature representation learning with momentum contrast (MoCo) for learning representation for histological and cytological images. Model training and evalutation were performed with PyTorch (version 1.12.1) on DGX A100 computing server. Source code for model development is publicly available at https://github.com/deeplearningplus/TORCH.

Data analysis

We develop a deep-learning-based method to assist pathologists in determining the origin of malignant hydrothorax and ascites using 57,220 cases of cytological smear images. We used four deep learning methods and trained a neural network model named TORCH while taking into account information from the entire package. Parameters including sex, age and specimen sampling site (hydrothorax and ascites) combined with cytological images were taken as inputs. We examined its performance on three internal testing sets (n=12799) and two external testing sets (n=14538). We compared TORCH with a panel of four pathologists on 496 cases that were randomly selected from testing sets. We trained our AI architecture in an end-to-end fashion with stochastic gradient descent for 100 epochs with a constant learning rate of 2e-4, weight decay of 1e-5 and batch size of 1 using Adam optimizer. Area under the receiver operating characteristic curve (AUROC) was used as the primary metric to measure classification performance. We conducted permutation test to determine whether there is statistically difference across five categories in terms of AUROC, precision and recall rate. The Clopper-Pearson method was used to calculate accuracy, sensitivity, specificity, positive predictive value, and negative predictive value. The Fleiss' kappa measures the inter-rater agreement among pathologists (R package irr, version 0.84). Rates of mortality were censored in September 2023 and calculated by Kaplan-Meier method. Log-rank test was employed to test the differences between Kaplan-Meier survival curves. Statistical analysis was performed by R software (version 3.9.1), pROC (version 1.17.0.1) and sklearn (version 0.24.1).

For manuscripts utilizing custom algorithms or software that are central to the research but not yet described in published literature, software must be made available to editors and reviewers. We strongly encourage code deposition in a community repository (e.g. GitHub). See the Nature Portfolio guidelines for submitting code & software for further information.

Data

Policy information about availability of data

All manuscripts must include a data availability statement. This statement should provide the following information, where applicable:

- Accession codes, unique identifiers, or web links for publicly available datasets
- A description of any restrictions on data availability
- For clinical datasets or third party data, please ensure that the statement adheres to our policy

Source code for model development is publicly available at https://github.com/deeplearningplus/TORCH. The TCGA whole-slide image data are available from NIH genomic data commons (https://portal.gdc.cancer.gov). The supporting data generated in this study are provided in the supplementary files. Sample data and cytological images for communication are given at DOI: https://doi.org/10.6084/m9.figshare.25270066. The fully treatment plan, survival information and other deidentified clinical data used in the treatment concordance analysis are available in Supplementary Table 17. Restrictions apply to the availability of cytological image data, which were used with institutional permission through IRB approval for the current study, and are thus not publicly available. Please email request for academic use of cytological image data to the corresponding author Xiangchun Li (lixiangchun@tmu.edu.cn) or first author Fei Tian (tianfei@tmu.edu.cn). All requests will be evaluated based on institutional and departmental policies to determine whether the data requested is subject to intellectual property or patient privacy obligations. Data can only be shared for non-commercial academic purposes and will require a formal material transfer agreement. Requests will be processed within 3 weeks.

Research involving human participants, their data, or biological material

Policy information about studies with human participants or human data. See also policy information about sex, gender (identity/presentation), and sexual orientation and race, ethnicity and racism.

Reporting on sex and gender

Our study is mainly focused on image related research, with sufficient research design and a large sample size. The ultimate dataset consisted of 57,220 images from 43,688 patients. We reported the sex distribution both in training set and five testing sets (see table 1). Additionally, among the overall 57,220 cases including both malignant and benign diseases, 45.1% are male and 54.9% are female, all of them are enrolled in a genuine manner. Meanwhile, our research findings are mainly based on the machine learning parameter results. Therefore, no sex- and gender-based analyses have been performed a priori. In the current study, sex was determined based on medical record identified from Chinese official issued identity ID card. Self-report gender was not used and reported in this study. We used biological sex assigned at birth as the clinical factor for TORCH and as confounding factor in survival analysis. We also included sex information in Supplementary Table 6, 16, 17.

Reporting on race, ethnicity, or other socially relevant groupings

57,220 cytological smear images from 43,688 patients were retrospectively or prospectively collected from cohorts of patients who attended four large Chinese tertiary referral institutions. No detailed race, ethnicity, or other socially relevant groupings were involved in this study. No socially relevant categorization variables were used in this study.

Population characteristics

Between June 2010 and October 2023, we obtained a large dataset of 90,572 cytological smear images of 76,183 patients from four large institutions (Tianjin Medical University Cancer Institute and Hospital, Zhengzhou University First Hospital, Suzhou University First Hospital, and Yantai Yuhuangding Hospital) as the training and testing sets (Table 1). We excluded 24,808 malignant images that did not have any clinical or pathological supporting evidence for the primary origins. Another 8,544 blank or blurry images were also excluded. The ultimate dataset consisted of 57,220 images from 43,688 patients. Of overall 57220 cases, 25822(45.1%) were male, 31398(54.9%) were female. The mean age was 59.13±14.21 years. 28079(49.1) cases were ≤60 years, 29141(50.9) cases were > 60 years. Training set consisted of 29,883 images from 20,638 individuals covering 12 tumor subtypes or origins: 138 esophagus images, 1,773 stomach images, 20 intestine images, 720 colon and rectum images, 151 liver images, 144 gallbladder, 357 pancreas images, 321 uterus and vagina images, 4,217 ovary and fallopian tube images, 1,874 breast images, 9,121 lung and upper respiratory tract images, and 570 blood-lymphatic system images. In addition to the 19,406 tumor images described above, 10,477 benign images were also included in ultimate training set. Similarly, three internal testing sets consisted of 10,974 individuals (12,799 images) from the same hospitals. Two additional external testing sets consisted of 12,076 individuals (14,538 images) from Tianjin and Yantai hospitals. The tumor category of testing sets was broadly in line with training set. As one patient might have more than once hydrothorax or ascites core needle biopsy for cytological analysis during different time of disease development, a patient could have more than one image. In this study, each image combined with its clinicopathological data was compiled as one case. Respiratory diseases accounted for the largest proportion (29.8%, n =17,058) among malignant groups. Carcinoma amounted to 56.7% (n=32,424) of overall hydrothorax and ascites cytological cases. Of which, the number of

	adenocarcinoma was the majority (47.2%, n=27,006). The proportion of squamous cell carcinoma metastasizing to pleural effusion or ascites was only 0.6% (n=346). Besides, there were 24,658 (82.5%) cases in the training set stratified as high-certainty, 5,225 (17.5%) as low-certainty. For testing sets, 18,184 (66.5%) cases in the training set were stratified as high-certainty, 9,153 (33.5%) as low-certainty. With respect to malignant images, 31.2% (6,066/19,406) cases of training set and 25.5% (4,256/16,702) cases of testing sets underwent sediment paraffin immunohistochemical staining examination additionally.
Recruitment	We retrospectively collected 42,682 cases of cytological smear images from cohorts of patients who attended three large tertiary referral institutions (Extended Data Fig. 3, Table1). Ultimately, we enrolled 14,008 cases from Tianjin Medical University Cancer Hospital between September 2012 and November 2020, 20,820 cases from Zhengzhou University First Hospital between August 2011 and December 2020, and 7,854 cases from Suzhou University First Hospital between June 2010 and December 2020. We randomly selected 70% of them as training sets and 30% as internal testing sets. We ensured that testing set patients did not overlap with those in the training set. Finally, the training sets consists of 29,883 cases and these three internal testing sets consists of 12,799 cases. For the convenience of description, we denoted these testing sets as Tianjin, Zhengzhou and Suzhou testing set, respectively. Particularly, we added two independent external testing sets that were enrolled from Tianjin Medical University Cancer Hospital between June 2023 and October 2023 (denoted as Tianjin-P testing set, 3,933 cases prospectively enrolled) and Yantai Yuhuangding General Hospital between February 2013 and May 2022 (denoted as Yantai testing set, 10,605 cases retrospectively enrolled). These two external testing sets are both fully unseen cohorts that were used further test the generalization capabilities of our model (Fig. 1).
Ethics oversight	Our work received approval from the institutional review board of Tianjin Medical University Cancer Institute and Hospital (IRB No.bc2021182). Data collection and other procedures were performed in accordance with principles of Good Clinical Practice and Declaration of Helsinki guidelines (1975, revised in 1983), as well as other relevant ethical regulations.

Note that full information on the approval of the study protocol must also be provided in the manuscript.

Field-specific reporting

Please select the one below that is the best fit for your research. If you are not sure, read the appropriate sections before making your selection.

☒ Life sciences ☐ Behavioural & social sciences ☐ Ecological, evolutionary & environmental sciences

For a reference copy of the document with all sections, see nature.com/documents/nr-reporting-summary-flat.pdf

Life sciences study design

All studies must disclose on these points even when the disclosure is negative.

Sample size	No specific statistical method for sample size calculation was performed. The sample size of our study was determined by the number of cases which can be curated retrospectively and data available at the time of data collection. Our purpose was to include as many patients/images as possible, as the performance of AI model was usually correlated with the number of training data. We used all the images retrieved from cytology imaging databases which belonged to four hospitals. Training set consisted of 29,883 images from 20,638 individuals covering 12 tumor subtypes or origins. Similarly, three internal testing sets consisted of 10,974 individuals (12,799 images) from the same hospitals. Two external testing sets consisted of 12076 individuals (14,538 images) from Tianjin and Yantai. The tumor category of testing sets was broadly in line with training set. Both training and testing sets contained a large dataset, which guaranteed sample sizes be sufficient.
Data exclusions	Between June 2010 and October 2023, we obtained a large dataset of 90,572 cytological smear images of 76,183 patients from four large institutions (Tianjin Medical University Cancer Institute and Hospital, Zhengzhou University First Hospital, Suzhou University First Hospital, and Yantai Yuhuangding Hospital) as the training and testing sets (Table 1). After case filtration and image quality control, we excluded 24,808 malignant images that did not have any clinical or pathological supporting evidence for the primary origins. Another 8,544 blank or blurry images were also excluded. The ultimate dataset consisted of 57,220 images from 43,688 patients.
Replication	We trained our model in an end-to-end fashion with stochastic gradient descent for 100 epochs with a constant learning rate of 2e-4, weight decay of 1e-5 and batch size of 1 using Adam optimizer. The findings were successfully validated in three internal testing sets and two external testing sets. The results showed that TORCH provided relatively reliable generalization and interoperability. The performance of TORCH was consistent across three internal testing sets (n=12799) and two external testing sets(n=14538). These five testing sets were from four different hospitals, with different population areas (Tianjin, Zhengzhou, Suzhou, Yantai), processing equipments (Olympus, Leica, Zeiss, Nikon), specimen sampling sites (Hydrothorax and ascites), application scopes (benign diseases and four common malignant categories). In both 495/496 cases study, performance comparision between TORCH and human pathologists revealed similar trend.
Randomization	42,682 cases of cytological smear images were retrospectively collected from cohorts of patients who attended three large tertiary referral institutions. After that, 70% of them were randomly selected as training set and 30% as testing set. To compare the performance of TORCH with that of experienced practicing pathologists, we randomly selected 495 cytological images from three internal testing sets for manual interpretation. Furthermore, to investigate whether junior pathologists' diagnostic ability could be improved or not by the assistance of TORCH, we randomly selected 496 additional cases (not overlapped with previous 495 cases) from three internal testing sets and presented prediction results from TORCH for these two pathologists for reference. For model development, we took into account clinical factors such as sex, age and tissue sampling sites. Confounding factors included in survival analysis are age, sex, TORCH-predicted cancer origin, tissue sampling site, number of metastatic sites and concordance of treatment.
Blinding	Patients' data were collected retrospectively or prospectively, and no form of single or double blinding was used during data collection process. We first retrieved pleural and peritoneal fluid isolated cells cytology data from pathology departments. To investigate whether our TORCH model could assist oncologists in tracing the origin of CUP patients and provide benefit for subsequent treatment, we retrospectively collected 391 uncertainty cases treated at Tianjin Cancer Hospital. Three senior clinical oncologists made comprehensive judgements

(concordant or discordant), they were blind to the follow-up information when making judgements.

Reporting for specific materials, systems and methods

We require information from authors about some types of materials, experimental systems and methods used in many studies. Here, indicate whether each material, system or method listed is relevant to your study. If you are not sure if a list item applies to your research, read the appropriate section before selecting a response.

Materials & experimental systems

n/a	Involved in the study
☒	☐ Antibodies
☒	☐ Eukaryotic cell lines
☒	☐ Palaeontology and archaeology
☒	☐ Animals and other organisms
☒	☐ Clinical data
☒	☐ Dual use research of concern
☒	☐ Plants

Methods

n/a	Involved in the study
☒	☐ ChIP-seq
☒	☐ Flow cytometry
☒	☐ MRI-based neuroimaging
